# Phylogenomic Evidence for a Myxococcal Contribution to the Mitochondrial Fatty Acid Beta-Oxidation

**DOI:** 10.1371/journal.pone.0021989

**Published:** 2011-07-07

**Authors:** Agatha Schlüter, Iñaki Ruiz-Trillo, Aurora Pujol

**Affiliations:** 1 Neurometabolic Diseases Laboratory, Institut d'Investigació Biomèdica de Bellvitge (IDIBELL), L'Hospitalet de Llobregat, Barcelona, Spain; 2 Institut de Neuropatologia, Hospital Universitari de Bellvitge, Universitat de Barcelona, Barcelona, Spain; 3 Centro de Investigación en Red sobre Enfermedades Raras (CIBERER), Valencia, Spain; 4 Departament de Genètica & Institut de Recerca en Biodiversitat (Irbio), Universitat de Barcelona, Barcelona, Spain; 5 Institució Catalana de Recerca i Estudis Avançats (ICREA), Barcelona, Spain; J. Craig Venter Institute, United States of America

## Abstract

**Background:**

The origin of eukaryotes remains a fundamental question in evolutionary biology. Although it is clear that eukaryotic genomes are a chimeric combination of genes of eubacterial and archaebacterial ancestry, the specific ancestry of most eubacterial genes is still unknown. The growing availability of microbial genomes offers the possibility of analyzing the ancestry of eukaryotic genomes and testing previous hypotheses on their origins.

**Methodology/Principal Findings:**

Here, we have applied a phylogenomic analysis to investigate a possible contribution of the Myxococcales to the first eukaryotes. We conducted a conservative pipeline with homologous sequence searches against a genomic sampling of 40 eukaryotic and 357 prokaryotic genomes. The phylogenetic reconstruction showed that several eukaryotic proteins traced to Myxococcales. Most of these proteins were associated with mitochondrial lipid intermediate pathways, particularly enzymes generating reducing equivalents with pivotal roles in fatty acid β-oxidation metabolism. Our data suggest that myxococcal species with the ability to oxidize fatty acids transferred several genes to eubacteria that eventually gave rise to the mitochondrial ancestor. Later, the eukaryotic nucleocytoplasmic lineage acquired those metabolic genes through endosymbiotic gene transfer.

**Conclusions/Significance:**

Our results support a prokaryotic origin, different from α-proteobacteria, for several mitochondrial genes. Our data reinforce a fluid prokaryotic chromosome model in which the mitochondrion appears to be an important entry point for myxococcal genes to enter eukaryotes.

## Introduction

The growing number of fully sequenced genomes from both prokaryotes and deep-branching eukaryotes offers the possibility of identifying genetic transfers that may have occurred when the first eukaryotes appeared at least 1.5 billion years ago [1]. Eukaryotic cells are chimeric entities, with both mitochondria and chloroplasts derived from endosymbiotic precursors that were distinct from the nucleocytoplasmic lineage [2]. Additionally, many of the original mitochondrially encoded genes were gradually transferred to the eukaryotic nuclear genome *via* endosymbiotic gene transfer [3]. It has been estimated that the α-proteobacterial ancestor of mitochondria contributed at least 630 genes to the eukaryotic nuclear genome [4]. However, thousands of eukaryotic nuclear genes of eubacterial ancestry are not derived from an α-proteobacterial ancestor [5], [6]. The hydrogen hypothesis proposes that the first eukaryote was a consortium of a hydrogen-dependent archaeon and a hydrogen-producing α-proteobacterium, which was the ancestor of the mitochondrion [7]. In this single eubacterial ancestry hypothesis, the mitochondrial ancestor would have assimilated other eubacterial genes via lateral gene transfer prior to the endosymbiotic event [5]. The fluid chromosome model would help to explain such mixed prokaryotic sources of the ancestral mitochondrial genome. This model assumes fluid prokaryotic genomes shaped by gene losses and lateral gene transfers instead of static genomes. Thus, the expected phylogeny for a gene acquired from the mitochondrion would be common ancestry for all eukaryotes but not necessarily trace to α-proteobacteria because the ancestor of mitochondria possessed an as yet unknown collection of genes. Alternatively, it has been recently demonstrated a high frequency gene transfer system in the α-proteobacteria *Rhodobacter capsulatus* based on a virus-like gene transfer agent (GTA), which would facilitate random transfers between species [8]. It has been suggested that the GTA system was present in the last α-proteobacteria common ancestor that could explain not only in as much so many α-proteobacterial genes came to reside in the nucleus, but also why mitochondrial and nuclear genes show such mixed phylogenetic affinities [9]. Several hypotheses have been suggested to account for more than a single (eubacterial) endosymbiont entity [5], [10]. One major hypothesis is metabolic symbiosis, also known as syntrophy, with a symbiont distinct from the ancestral mitochondrion symbiont. One syntrophy case is based upon the exchange of sulfur compounds between a spirochete and an archaeal thermoplasma species [11]. Another case is the hydrogen-driven syntrophy hypothesis, which suggests that the eukaryotic ancestor was derived from symbiosis between an ancestral δ-proteobacteria, specifically a sulfate-reducing myxococcal species, and a methanogenic archaeon followed by the eventual incorporation of an α-proteobacterium. The latter hypothesis argues for the full incorporation of a methanogenic archaebacterium within a myxococcal cytoplasm. The acquisition of mitochondria was independent and early in the existing myxococcal-archaeon consortia [12], [13]

The δ-proteobacteria represent one of the most diverse groups of bacteria, exhibiting a wide array of metabolic strategies, including free-living, syntrophic and pathogenic forms. This group is characterized by large variations in genome size. While species of the genus *Syntrophus* have genomes of approximately 3 Mb, the genomes of myxococcal species are among the largest in prokaryotes, with sizes of approximately 9–13 Mb, which might account for their higher biological complexity [14]. The Myxococcales are characterized by social behavior directed toward predation and the construction of a unique multicellular structure, the asexual fruiting body [15]. The formation of these fruiting bodies requires coordinated cellular motility and cell signaling [15]. Thus, the Myxococcales are of special interest because they represent one of the few prokaryotic lineages that have independently acquired some degree of cellular differentiation and multicellularity. In the Myxococcales, lipids play a role in developmental aggregation, signaling and morphogenesis of the fruiting body [15]. In addition, lipids are major contributors to myxococcal physiology as an energy reservoir, which is also the case in fungi, plants and animals, but not in most other prokaryotes [16]. The Myxococcales have also been shown to share other similarities with eukaryotes, such as the presence of eukaryotic-like protein kinases [17], Ras-like G-proteins and GTPase-activating proteins that function in regulating cell polarity [18]. Here, we employed a phylogenomic analysis to investigate a possible contribution of Myxococcales to the origin of eukaryotes. Our data revealed that several genes encoding mitochondrial proteins, mostly from the fatty acid β-oxidation pathway, were acquired from Myxococcales.

## Results

### Phylogenetic analysis

**Figure 1:**
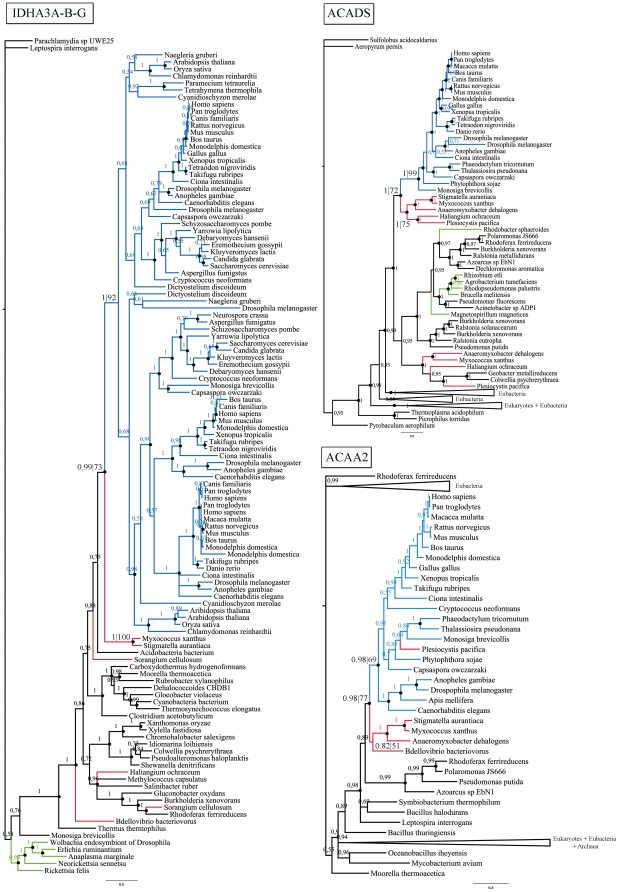
Myxococcal ancestry of eukaryotic proteins. Bayesian phylogenetic trees of (i) the isocitrate dehydrogenase 3 NAD(+) alpha, beta and gamma genes, (ii) the acyl-CoA dehydrogenase C-2 to C-3 short chain gene (ACADS), and (iii) acetyl-CoA acyltransferase 2 (ACAA2). The Bayesian posterior probability (PP) until convergence diagnostic and 1000-replicate bootstrap values (BV) for ML trees are indicated if they were above 50%. A black dot indicates PP>0.95. Eukaryotic, myxococcal/δ-proteobacterial and α-proteobacterial taxa are highlighted in blue, red and green, respectively.

To identify putative genetic transfers between eukaryotes and Myxococcales, we conducted a large-scale comparative genomic analysis of 40 eukaryotic genomes, 27 archaeal genomes and 330 eubacterial genomes, including 6 myxococcal species: *Anaeromyxobacter dehalogenans*, *Haliangium ochraceum*, *Myxococcus xanthus*, *Plesiocystis pacifica*, *Sorangium cellulosum* and *Stigmatella aurantiaca*. A preliminary and conservative step consisting of BLAST (threshold E-value of e-40) homologous sequence searches and maximum likelihood (ML) phylogenetic tree reconstruction identified 93 eukaryotic proteins with a predicted myxococcal origin (Figure S1) followed by 40 trees with α-proteobacterial, 15 with archaeal, 8 with firmicutes, 7 with cyanobacterial and 7 with chlamydial origin. The remaining trees traced to other bacterial groups but had less than four trees or were unresolved, with several prokaryotic groups preceding eukaryotes. These 93 positives were further evaluated using HMMER searches, a tool based on hidden Markov models, against additional eukaryotic genomes, and the alignments were performed again with these new taxa. From the inferred ML and Bayesian trees, we identified 15 eukaryotic proteins of obvious myxococcal origin, all with strong or moderate statistical support (Figure 1; see Dataset S1 for all ML trees and Dataset S2 for all Bayesian trees).

**Figure 2:**
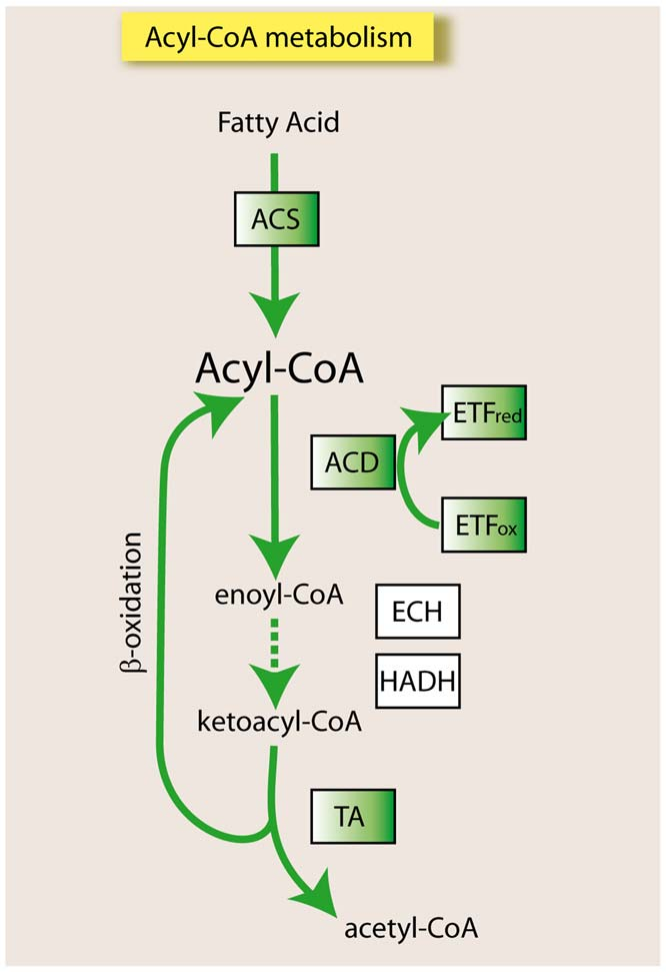
The acyl-CoA pathway. Schematic representation of the acyl-CoA pathway, including the β-oxidation cycle. Enzymes with myxococcal ancestry are indicated in green boxes. Abbreviations: ACS, acyl-CoA synthetase; ACD, acyl-CoA dehydrogenase; ETF, electron transport flavoprotein; ECH, enoyl-CoA hydratase; HADH, hydroxyacyl-CoA dehydrogenase; and TA, thioesterase.

Of note, 13 of these 15 genes of myxococcal ancestry were localized to the mitochondria in eukaryotes (see Table 1). We determined the organellar localization of these genes based on bibliographic references and by detecting predicted mitochondrial targeting sequences for several eukaryotes (Table 1). Eight of these proteins play a role in the formation of the acyl-CoA pool; which comprises pivotal intermediates in lipid metabolism (Figure 2). In particular, we detected a myxococcal ancestry for two eukaryotic acyl-CoA synthetases (ACSs) and, remarkably, six fatty acid β-oxidation enzymes that degrade the acyl-CoA pool: four acyl-CoA dehydrogenases (ACDs), one electron transport flavoprotein (ETF) and one acetyl-CoA acyltransferase with thiolase activity (see Table 1). The ACSs identified corresponded to ACS bubblegum family members 1 and 2 (ACSBG1–ACSBG2) and ACS family member 3 (ACSF3), which activates fatty acids to form acyl-CoA, thus allowing their transport and metabolism [19]. The ACD protein family catalyzes the oxidation of diverse acyl-CoA compounds produced during the degradation of fat and protein to enoyl-CoA [20]. ACD subfamilies are distinguished by the metabolic pathways in which they participate and by their substrate specificity. The four eukaryotic ACDs with myxococcal ancestry participate in the β-oxidation of fatty acids, with optimal activity for acyl-CoA substrates of specific lengths: short (ACADS), medium (ACADM), long unsaturated (ACAD9) or very long (ACADVL) [20], [21]. The other ACD subfamily identified is implicated in amino acid degradation. After removal of the amino groups from isoleucine, the remaining branched acyl-CoA is dehydrogenated by the short/branched chain acyl-CoA dehydrogenase (ACADSB). Electron transport flavoprotein A (ETFA) is, in addition to electron transport flavoprotein B (ETFB), the primary acceptor for reducing equivalents from the β-oxidation of acyl-CoA dehydrogenases [22]. The phylogenetic trees of both electron transport flavoproteins (ETFA and ETFB) showed patchy distributions, with eukaryotes nested within different bacterial groups; however, the bacterial group first identified as preceding eukaryotes corresponded to the Myxococcales (see ETFA tree in Dataset S1 and S2). Remarkably, an acyl-CoA dehydrogenase gene (ACADM) is located next to the ETFA and ETFB genes in the genome of *M. xanthus*, indicating the importance of electron flow during the β-oxidation of acyl-CoA intermediates in *M. xanthus*. Moreover, the thiolase identified, acetyl-CoA acyltransferase 2 (ACAA2), performs the last thiolytic cleavage of the fatty acid β-oxidation spiral in mitochondria and it should be differentiated from the peroxisomal thiolase ACAA1 and from the thiolases ACAT1 and ACAT2 that can also act in the biosynthesis of eukaryotic ketone bodies and sterols through the condensation of two acetyl-CoA molecules to form acetoacetyl-CoA [23]. The ACAA2 thiolase has previously been reported to have a myxococcal origin [24].

**Table 1:** List of eukaryotic proteins with a predicted myxococcal origin.

Biological function			Symbol	Support (BV/PP)	Localization	Mitochondria-targeting signal peptide
Lipid metabolism	Acyl-CoA synthetase		ACSF3	100/1	Mitochondria	*Aspergillus*, *Capsaspora*, Metazoa, Trypanosomatids
Lipid metabolism	Acyl-CoA synthetase (EC:6.2.1.3)		ACSBG1ACSBG2	56/0.57	Cytosol	none
Lipid metabolism	Fatty-acid β-oxidation	Acyl-CoA dehydrogenase (EC:1.3.99.3)	ACADM	70/0.84	Mitochondria	*Capsaspora*, Metazoa, *Phytopthora*, Trypanosomatids
Lipid metabolism	Fatty-acid β-oxidation	Acyl-CoA dehydrogenase (EC:1.3.99.2)	ACADS	72/1	Mitochondria	*Capsaspora*, Metazoa
Lipid metabolism	Fatty-acid β-oxidation	Acyl-CoA dehydrogenase (EC:1.3.99.12)	ACADSB	78/1	Mitochondria	*Capsaspora*, *Cyanidioschyzon*, Fungi, Metazoa, *Phaeodactylum*, Trypanosomatids
Lipid metabolism	Fatty-acid β-oxidation	Acyl-CoA dehydrogenase (EC:1.3.99.-)	ACADVL ACAD9	96/0.98	Mitochondria	*Capsaspora*, Metazoa
Lipid metabolism	Fatty-acid β-oxidation	Electron-transfer- flavoprotein	ETFA	95/0.99	Mitochondria	Archaeplastida, *Capsaspora*, *Dictyostelium*, Excavata, Metazoa
Lipid metabolism	Fatty-acid β-oxidation	Acyl-CoA acyltransferase (EC:2.3.1.16)	ACAA2	77/0.98	Mitochondria	*Capsaspora*, Fungi, Metazoa
Lipid metabolism	Ceramidase (EC:3.5.1.23)	ASAH2	100/0.91	Mitochondria	none	
Proteases	Metallopeptidase M3 family (EC:3.4.24.15/3.4.24.16)	NLN THOP1	100/1	Mitochondria and cytosol	Archaeplastida, *Capsaspora*, Fungi, Metazoa, Trypanosomatids	
Proteases	Aminopeptidase (EC:3.4.11.-)	NPEPL1	100/1	???	none	
Amino acid metabolism	Aminobutyrate aminotransferase (EC:2.6.1.19/2.6.1.22)	ABAT	95/1	Mitochondria	*Capsaspora*, *Dictyostelium*, Fungi, Metazoa	
Translational system	Translation elongation factor	GFM1	92/0.66	Mitochondria	*Capsaspora*, Metazoa, Trypanosomatids	
TCA cycle	NAD(+) dependent isocitrate dehydrogenase (EC:1.1.1.41)	IDHA3A IDHA3B IDHA3G	73/0.99	Mitochondria	Archaeplastida, *Capsaspora*, *Dictyostelium*, Fungi, Metazoa	
*tRNA synthesis*	Arginyl tRNA synthetase (EC:6.1.1.19)	RARS2	75/0.99	Mitochondria	*Capsaspora*, Fungi, Metazoa	

Symbol corresponds to the human annotation as found in NCBI. Support indicates the statistical support for a monophyletic myxococcal+eukaryotic group as obtained from the 1000-replicate ML bootstrap values (BV, in %) and the Bayesian posterior probability (PP). Localization indicates the localization determined for most of the studied eukaryotes. Taxa shown in the last column are the eukaryotes in which the TargetP program (http://www.cbs.dtu.dk/services/TargetP/) [52] detected a putative mitochondria-targeting peptide. When generic lineage names, such as Archaeplastida, Excavata, Fungi, Metazoa or Trypanosomatids, are shown, at least two different species of this group were found to have a predicted signal peptide.

The remaining proteins of unequivocal myxococcal ancestry belonged to different functional groups, and five of them also localized to the mitochondria (Table 1). These remaining proteins are as follows:

- three proteases: the M3 family peptidases neurolysin (NLN) and the thimet oligopeptidase (THOP1) and the cytosolic aminopeptidase NPEPL1;
- a translation elongation factor G that is predicted to localize to mitochondria in organisms ranging from mammals to trypanosomatids and that performs GTP-dependent translocation of the ribosome during translation;
- the Krebs cycle enzyme NAD(+)-dependent isocitrate dehydrogenase;
- an arginil tRNA synthetase;
- an aminotransferase, 4-aminobutyrate aminotransferase; and
- a ceramidase, N-acylsphingosine amidohydrolase 2, that catalyzes the hydrolysis of the N-acyl linkage of ceramide, a second messenger in a variety of cellular events, to produce sphingosine. Despite the lack of a predicted mitochondrial targeting sequence, this protein has been experimentally localized to the mitochondria of mice and humans [25], [26].

### Compositional analysis

The position of the Myxococcales as a sister group to eukaryotes in phylogenetic trees produced in this study implies the occurrence of lateral gene transfer events from Myxococcales to eukaryotes or vice versa (Figure 1). We investigated this possibility further by performing a sequence compositional analysis of the 15 eukaryotic genes of myxococcal ancestry using homologs from both Myxococcales and unicellular eukaryotes [27]. In cases of recent lateral gene transfer, it has been suggested that the nucleotide composition of the transferred gene might be more similar to that of the donor species than to that of the recipient [28]. The average G+C content of myxococcal species ranges from 69 to 74%. To examine the possibility of a transfer from Myxococcales to eukaryotes, we calculated the average G+C content in the coding sequence (CDS) of our 15 candidate genes in the unicellular eukaryotes and myxococcal homologs and compared them to the average whole-genome G+C content for each species. We found that the 15 CDSs from unicellular eukaryotes were consistently representative of their respective genomes and were thus adapted to function in the organism in which they resided, an observation that was inconsistent with the occurrence of recent lateral gene transfer events.

To further examine the possibility of a eukaryote-to-Myxococcales transfer, we used several compositional methods to measure lateral gene transfer in bacteria: (i) Karlin's method based on codon usage [29], [30], (ii) the Horizontal Gene Transfer Database (HGT-DB), which relies on G+C content, codon usage, gene position and amino acid composition [31], and (iii) the Island Viewer server, which identifies genomic islands or clusters by integrating sequence composition and comparative genomic approaches. None of the 15 myxococcal ancestors of eukaryotic genes was predicted to have undergone lateral gene transfer from eukaryotes. This analysis only identified overlaps of 3 and 6 bp in ACADS and ACSBG, respectively, in the *M. xanthus* genome (Figure S2). Thus, our findings on the base composition of the 15 CDS candidates are not consistent with recent lateral gene transfers. Instead, our results suggest that ancestral genetic transfers occurred between Myxococcales and eukaryotes, and the incorporated genes might have since converged with the bulk of the genome through a process of amelioration [32].

## Discussion

### Myxococcal origin of part of the mitochondrial β-oxidation pathway

Although we used a conservative pipeline (threshold E-value of e-40) and despite few myxococcal genomes being currently available, our results clearly indicate that several eukaryotic genes have a myxococcal ancestry. Half of the genes identified encoded mitochondrial enzymes involved in acyl–CoA intermediate metabolism, primarily in fatty acid β-oxidation (Table 1 and Figure 2). Among the enzymes involved in β-oxidation with a clear myxococcal ancestry were four acyl-CoA dehydrogenases (ACDs), which catalyze the oxidation of diverse acyl-CoA compounds produced during the degradation of fat and amino acid to enoyl-CoA in a substrate-specific manner [20]. Fatty acids can be degraded in hydrogen-driven syntrophy with symbiotic partners under anaerobic conditions [33], [34]. There are natural examples of fatty acid syntrophies with δ-proteobacteria. For instance, the fermenting *Syntrophaceae* family has the ability to grow on fatty acids in syntrophy with methanogens [34], [35]. Notably, the syntrophic oxidation of fatty acids involves hydrogen production from high potential electron donors, such as the acyl-CoA intermediates, that are oxidized by members of the ACD family [36]. Indeed, a genomic analysis of the fatty acid degrading syntrophic bacterium *Syntrophus aciditrophicus* has suggested that the oxidation of acyl-CoA intermediates, which plays a crucial role in generating reducing equivalents, might be specific to syntrophic metabolism [33]. The production of hydrogen from the ACD reaction is thermodynamically unfavorable and can occur only with energy input by a process known as reverse electron transfer. With regard to this mechanism, it has been suggested that ETF could transfer electrons from acyl-CoA intermediates oxidized by ACD to membrane redox complexes, such as the complex that includes the membrane-bound iron-sulfur oxidoreductase present in *S. aciditrophicus* [33], [36].

Our list of putative fatty acid β-oxidation genes with a myxococcal ancestry did not include the genes encoding the central multifunctional proteins involved in β-oxidation (HADHA and HADHB). A possible explanation for this absence may lie in the fact that the human HADHA/HADHB multienzyme complex is formed by a gene fusion between 3-hydroxyacyl-CoA dehydrogenase and enoyl-CoA hydratase of HADHA and the non-covalent interaction of the thiolase activity of HADHB. This arrangement probably resulted from the combination of monospecific enzymatic functions [37]. Thus, the gene fusion present in the human genome from which we retrieved myxococcal homologous sequences (step 1 in our pipeline) might have masked ancestral monofunctional subunits that are still functional in other eukaryote lineages (for example, in *Euglena gracilis* [37]). Indeed, we identified a monofunctional enzyme with 3-hydroxyacyl-CoA dehydrogenase activity with myxococcal ancestry, although the statistical support for this inference was low (38% ML and 0.91 BPP). This protein is absent in Metazoa, but *Capsaspora*, Chromalveolata, *Dictyostelium*, *Naegleria* and Fungi all encode homologs of this gene (Figure S3). Our results contradict those of studies suggesting that the α-proteobacterial ancestor of mitochondria was the donor of multiple genes involved in acyl-CoA metabolism, including the β-oxidation pathway, to the nucleocytoplasmic lineage [4]. This is most likely because our analysis involved a higher number of myxococcal genomes and included a relatively large taxon sampling. However, we cannot exclude the possibility that the α-proteobacterial ancestor of mitochondria was also a fatty acid degrading bacteria [38], with the possibility that some eukaryotic β-oxidation enzymes could have been acquired from α-proteobacterial genomes. Indeed, our results indicate that some mitochondrial β-oxidation enzymes did not descend from α-proteobacterial homologs because they were absent in the reported trees (see the ACADSB tree in Figure 2 and Dataset S1 and S2) or their topology was unrelated to the eukaryotic clade (see ACAA2, ACADM, ACADS, ACADVL-ACAD9 and ACSBG trees in Figure 2 and Dataset S1 and S2).

### Lateral gene transfer from Myxococcales to eukaryotes or fluid chromosome?

Our results are compatible with the occurrence of multiple ancient lateral gene transfer events from Myxococcales to eukaryotes. We propose three possible explanations for the observation that most of the proteins with myxococcal ancestry found in this study are targeted to the mitochondria. First, myxococcal bacteria may have transferred genes to the nucleocytoplasmic lineage using direct and independent lateral gene transfers. Second, lateral transfer may have occurred *via* endosymbiotic gene transfer from a myxococcal symbiont to the nucleocytoplasmic lineage. This second proposal is compatible with the δ-proteobacteria syntrophy hypothesis, which suggests that the eukaryotic ancestor was derived from a symbiosis between an ancestral sulfate-reducing myxococcal species and a methanogenic archaeon [12], [13]. However, this hypothesis assumes an unrelated incorporation of the myxococcal and mitochondrial ancestors, which does not support the majority of the myxococcal ancestry proteins found in this study being targeted to the mitochondria. The δ-proteobacteria syntrophy hypothesis argues that a myxococcal endosymbiosis occurred prior and independently of the mitochondrion acquisition by an amitochondriate eukaryote. We believe that our findings are more compatible with a simultaneous origin for myxococcal and mitochondria proteins that might underlie the reason why proteins with myxococcal origin are preferably targeted to the mitochondrion. A third possibility is that ancestral lateral gene transfer events occurred between Myxococcales and the mitochondrial ancestor endosymbiont prior to the endosymbiotic event that gave rise to the mitochondrion. In this latter scenario, the myxococcal genes would have already been present in the ancestral mitochondrial genome. This option is compatible with the fluid chromosome model [39], [40]. Thus, myxococcal transfers to eukaryotic genomes would have originated vertically from the mitochondrial progenitor, which was not necessarily an α-proteobacterium because the first mitochondrial genome possessed an as yet unknown collection of genes.

Taken together, we believe that the most parsimonious event might have been that a hydrogen-producer, a fatty acid degrading δ-proteobacteria, transferred acyl-CoA related enzymes to the hydrogen producing mitochondrial ancestor (Figure 3.1). According to the hydrogen hypothesis on the origin of eukaryotes, eukaryotes arose from a hydrogen-driven syntrophy between the hydrogen producing α-proteobacteria symbiont and a hydrogen-consuming methanogenic archaeon [7]. In this scenario, harboring several ACD reducing equivalents producers acquired from Myxococcales could be of advantage for the hydrogen producing mitochondrial ancestor, fostering hydrogen, acetate and CO_2_ production. Once the host methane production became irreversibly dependent on the symbiont hydrogen source, the methanogenic archaebacterium would have maximized its contact with the surface area of the symbiont by surrounding it, eventually engulfing the hydrogen producing symbiont. Over time, genes would have been transferred through endosymbiotic gene transfer to the eukaryotic nucleocytoplasmic lineage (Figure 3.2). If this scenario is true, we would expect that at least some mitochondrially encoded genes would trace to Myxococcales. To test this possibility, we analyzed the myxococcal ancestry of mitochondrially encoded proteins from 2209 mitochondrial genomes. Homologous sequence searches and subsequent phylogenetic reconstruction indicated that none of the mitochondrially encoded proteins had a myxococcal origin (data not shown). Taking into account this result, we cannot discard the possibility of a myxococcal endosymbiosis. The lack of myxococcal ancestry in mitochondrially encoded proteins might apparently support an independent myxococcal endosymbiotic event, but then the genes transferred to the host should be targeted not only to the mitochondrion but colonizing other subcellular compartments. As far as the myxococcal proteins identified are preferably targeted to the mitochondria, we believe that the most compatible scenario is the simultaneous origin for mitochondrial and myxococcal proteins. According to that, there are two possibilities: i) that the endosymbiotic event that gave rise to the mitochondrion also involved a myxoccocal bacterial partner that subsequently transferred a number of metabolic pathways to the host via endosymbiotic gene transfer; or ii) ancestral lateral gene transfer events took place between Myxococcales and the mitochondrial ancestor endosymbiont prior to the endosymbiotic event that gave rise to the mitochondrion. Supporting the last option, the mitochondrial genome seems to retain specific functional genes that do not descend from the myxococcal genomes. In this sense, the presence of a mitochondrial genome appears to correlate with increased respiratory capacity and ATP availability within organisms [41], [42]. Therefore, it has been suggested that this relationship could be the driving force behind the selective pressure to preferentially retain the genes encoding the electron transport chain in the mitochondrial genome [41], [42]. These genes are mostly derived from α-proteobacterial genomes; in contrast, the myxobacterially derived metabolic genes would have been transferred to the eukaryotic nucleocytoplasmic lineage. This transfer might account for the absence of mitochondrially encoded genes with myxococcal ancestry.

**Figure 3:**
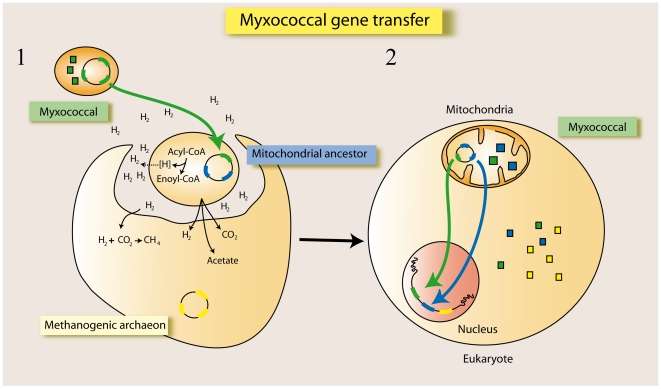
Model of transfer of myxococcal genes to eukaryotes. (1) A hydrogen-consuming methanogenic archaeon established symbiosis with the hydrogen-producing mitochondrial ancestor. Previously, a fatty acid degrading myxococcal species transferred genes to the mitochondrial ancestor before the endosymbiotic event. (2) The methanogenic archaeon engulfed the symbiont that gave rise to the mitochondrion. Over time, genes were transferred to the eukaryotic nuclear genome via endosymbiotic gene transfer. Proteins with myxococcal, mitochondrial and archaeal ancestries are depicted in green, blue and yellow boxes, respectively.

It is worth mentioning that because Myxococcales harbors some of the largest eubacterial genomes, lineage sorting could influence our topologies. However, most of our trees have representation from a wide sampling of other eubacterial genomes. Our results indicate that the mitochondrion might be an important entry point of eubacterial genes that are different from α-proteobacterial genes. Thus, the genome of the mitochondrial ancestor should be considered a mix of different eubacterial genes, some of them still conserved in the extant α-proteobacterial and myxococcal genomes. Further, it is tempting to speculate that the large genomes of the Myxococcales could be a repository of ancestral genes from many prokaryotic lineages, including that of the mitochondrial ancestor.

In conclusion, our data indicate the myxococcal origin of 15 nuclear eukaryotic genes that are not α-proteobacterial, some of which have key roles in acyl-CoA intermediate metabolism. Many other genes may also have a myxococcal origin, but the lack of a phylogenetic signal and/or our extremely conservative pipeline did not allow us to recover them. We propose that a fatty acid degrading δ-proteobacterium donated some genes to the mitochondrial ancestor prior to the endosymbiotic event, and these genes were subsequently transferred to the nucleocytoplasmic lineage via endosymbiotic gene transfer. Thus, our results support a version of the fluid chromosome model as the most plausible scenario, in which Myxococcales contributed key metabolic genes to the first eukaryotes.

## Materials and Methods

### Data retrieval

Proteomes encoded by 355 publicly available complete genomes of eubacteria and archaea were obtained from the National Center for Biotechnology Information (NCBI) FTP server (ftp://ftp.ncbi.nih.gov/genomes/Bacteria), including four complete myxococcal genomes. In addition, we included the draft assembly genomes of the Myxococcales species *P. pacifica* and *S. aurantiaca*. We sampled 40 and 49 eukaryotic genomes for the first and second analyses, respectively, with the goal of encompassing the widest possible eukaryotic diversity. Sequence analyses were performed for a range of members of the Amoebozoa (*Dictyostelium discoideum*), the Archaeplastida (*Arabidopsis thaliana*, *Chlamydomonas reinhardtii*, *Cyanidioschyzon merolae*, *Oryza sativa* and *Ostreococcus tauri*), the Chromalveolata (*Paramecium tetraurelia*, *Phaeodactylum tricornutum*, *Phytophthora sojae*, *Tetrahymena thermophila*, *Theileria parva* and *Thalassiosira pseudonana*), the Excavata (*Giardia lamblia*, *Leishmania major*, *Naegleria gruberi*, *Trichomonas vaginalis*, and *Trypanosoma brucei and cruzi*), and the Opisthokonta (*Anopheles gambiae*, *Apis mellifera*, *Aspergillus aspergillus*, *Bos taurus*, *Caenorhabditis elegans*, *Candida glabrata*, *Canis familiaris*, *Capsaspora owczarzaki*, *Ciona intestinalis*, *Cryptococcus neoformans*, *Danio rerio*, *Debaryomyces hansenii*, *Drosophila melanogaster*, *Gallus gallus*, *Homo sapiens*, *Encephalitozoon cuniculi*, *Eremothecium gossypii*, *Kluyveromyces lactis*, *Macaca mulatta*, *Monodelphis domestica*, *Monosiga brevicollis*, *Mus musculus*, *Neurospora crassa*, *Pan troglodytes*, *Rattus norvegiccus*, *Takifugu rubripes*, *Tetraodon nigroviridis*, *Saccharomyces cerevisiae*, *Schizosaccharomyces pombe*, *Xenopus tropicalis* and *Yarrowia lipolytica*).

The databases used included the ENSEMBL databases for *A. gambiae*, *A. mellifera*, *B. taurus*, *C. elegans*, *C. familiaris*, *C. intestinalis*, *D. rerio*, *D. melanogaster*, *G. gallus*, *H. sapiens*, *M. mulatta*, *M. domestica*, *M. musculus*, *P. troglodytes*, *R. norvegiccus*, *S. cerevisiae*, *T. rubripes*, *T. nigroviridis* and *X. tropicalis*; the NCBI genomes for *A. fumigatus*, *C. glabrata*, *C. neoformans*, D. hansenii, *E. cuniculi*, *E. gossypii*, *K. lactis*, *L. major*, *N. crassa*, *O. sativa*, *S. pombe*, *T. thermophila*, *T. brucei*, *T. cruzi*, *T parva* and *Y. lipolytica*; the *Cyanidioschyzon merolae* Genome Project (http://merolae.biol.s.u-tokyo.ac.jp) for *C. merolae*; DictyBase (http://dictybase.org) for *D. discoideum*; GiardiaDB (http://giardiadb.org) for *G. lamblia*; the Integr8 Database for *A thaliana*; TrichDB (http://trichdb.org) for *T. vaginalis*; and the Joint Genome Institute Eukaryotic Genomics databases (http://genome.jgi-psf.org) for *T. pseudonana, N. gruberi, P. sojae, P. tricornutum, O. tauri, M. brevicollis, C. reinhardtii, P. tetraurelia and C. owczarzaki*. For the second analysis, we included the following genomes: *N. gruberi*, *P. sojae*, *P. tricornutum*, *O. tauri*, *M. brevicollis*, *C. reinhardtii*, *P. tetraurelia*, *C. owczarzaki* and *O. sativa*.

### Homolog sequence searches and phylogenetic reconstruction

For the complete Myxococcales genomes and the *P. pacifica* proteome, homologous sequences were retrieved using blastx and blastp algorithms (E<10^−40^), respectively, against the human proteome. This search yielded a set of 471 human proteins that were used as seed proteins to identify homologous proteins against the ensemble of 357 bacterial and 40 eukaryotic genomes described above (E<10^−40^). Groups of homologous sequences for each of the 471 seed proteins were aligned using MAFFT with default parameters [43]. Gap rich positions in the alignment were removed using trimAl v1.2, applying a gap threshold of 25% and a conservation threshold of 50% [44]. Maximum likelihood (ML) phylogenetic trees were then reconstructed in RAxML 7.0.4 [45] using the Whelan and Goldman (WAG) matrix of amino acid replacements and assuming a proportion of invariant positions (WAG+I). The number of bootstrapping runs was automatically determined using a newly implemented rapid bootstrap algorithm for RAxML [45] using CIPRES-Portal 2.0 [46]. The resulting 471 ML trees were examined, yielding 93 trees with a eukaryotic cluster that putatively branched with a myxococcal clade. For each of the resulting 93 trees, we built the eukaryotic protein profile using a tool based on hidden Markov models, HMMER version 3.0 (http://hmmer.org) [47]. The eukaryotic proteins from these selected trees were used to repeat the searches with HMMER on a proteome dataset including 357 bacterial and archaeal proteomes and 49 eukaryotic proteomes using a cutoff range of E<10^−3^. Groups of homologous sequences were aligned using MAFFT. Insertions and sequence characters that could not be aligned with confidence and incomplete sequences were removed. Multi-aligned sequences were manually examined. Additional phylogenetic analyses were performed using both RAxML and MrBayes [48] analyses. One thousand replicates of rapid bootstrap analyses were performed using RAxML 7.2.6 with the general time-reversible (GTR) matrix of amino acid replacements and four gamma-distributed rates (GTR+Г) using CIPRES-Portal 2.0 [46]. Additional phylogenetic analyses were performed using a Bayesian method implemented in MrBayes with a mixed model of amino acid substitution and with gamma correction, including four discrete correction categories and a proportion of invariant sites (WAG+Г+I). MrBayes was run until a convergence diagnostic with a standard deviation of split frequencies <0.01 was achieved or until the likelihood of the cold chain stopped increasing and began to randomly fluctuate, thus reaching a stationary state. The analyses were performed with eight chains, and trees were sampled every 1000 generations in two runs. To construct the consensus tree, the first 10% of trees were discarded. When necessary, large unresolved trees were split into smaller partitions with strong bootstrap support or differentiated long branches to facilitate the analysis.

### Mitochondrial proteome analysis

Mitochondrial proteomes were retrieved from 2209 eukaryotes from http://www.ncbi.nlm.nih.gov/genomes/GenomesGroup.cgi?opt=organelle&taxid=2759. Approximately 30000 proteins were grouped into 53 orthologous groups, and homologous BLAST searches were performed against 357 bacterial and archaeal proteomes (E<10^−20^). Phylogenetic reconstruction revealed only 12 potential myxococcal ancestries, which were then discarded based on HMM searches (E<10^−3^) and subsequent ML and Bayesian phylogenetic reconstructions.

### Lateral gene transfer predictions

We analyzed the sequence composition of the fully sequenced myxococcal genomes of *A. dehalogenans*, *H. ochraceum*, *M. xanthus*, and *S. cellulosum* using three different methods: (i) Karlin's method based on determined codon usage and calculated using software available from the Computational Microbiology Laboratory (http://www.cmbl.uga.edu/software.html) [29], [30], (ii) the Horizontal Gene Transfer Database (HGT-DB), which relies on G+C content, codon usage, gene position and amino acid content (http://genomes.urv.es/HGT-DB/) [31], and (iii) the Island Viewer server to identify genomic islands or clusters, which integrates sequence composition and comparative genomics approaches (http://www.pathogenomics.sfu.ca/islandviewer) [49]. In supplementary figures, the open reading frames (ORFs) were depicted using CGView [50]. Codon usage tables and G+C content values from eukaryotes and Myxococcales were extracted from the Codon Usage Database (http://www.kazusa.or.jp/codon/).

## Supporting Information

Figure S1 Schematic diagram of the automatic pipeline followed to identify eukaryotic proteins with a predicted myxococcal origin.
(TIF)

Figure S2 Circular genome maps of *A. dehalogenans*, *H. ochraceum*, *M xanthus* and *S. cellulosum*, showing their respective ORFs. In the outermost layer of the circle, the proposed myxoccocal ancestor genes are indicated as black stripes. The abbreviated names of the corresponding eukaryotic proteins are indicated in parentheses. Putative gene acquisitions via lateral gene transfer are displayed with three different methods: a) alien genes via the Karlin method in red, b) lateral gene transfers from HGT-DB in green, and c) genomic islands using the Island Viewer server in yellow.
(PDF)

Figure S3 ML and Bayesian phylogenetic tree of a monofunctional hydroxyacyl-CoA dehydrogenase (HADH) protein. Eukaryotic, myxococcal/δ-proteobacterial and α-proteobacterial taxa are highlighted in blue, red and green, respectively.
(PDF)

Dataset S1 Maximum likelihood phylogenetic trees of the 15 eukaryotic proteins that branch with a myxococcal clade. The eukaryotic and myxococcal clades are highlighted in blue and red, respectively. The 1000-replicate ML bootstrap values are shown in the branches.
(PDF)

Dataset S2 Bayesian phylogenetic trees of the 15 eukaryotic proteins that branch with a myxococcal clade. The eukaryotic and myxococcal clades are highlighted in blue and red, respectively.
(PDF)
