# Supplementary figures and images for: Phylogenomic Evidence for a Myxococcal Contribution to the Mitochondrial Fatty Acid Beta-Oxidation
Source: PLoS One. 2011 Jul 7;6(7):e21989. doi: 10.1371/journal.pone.0021989 (PMC3131387; doi:10.1371/journal.pone.0021989)

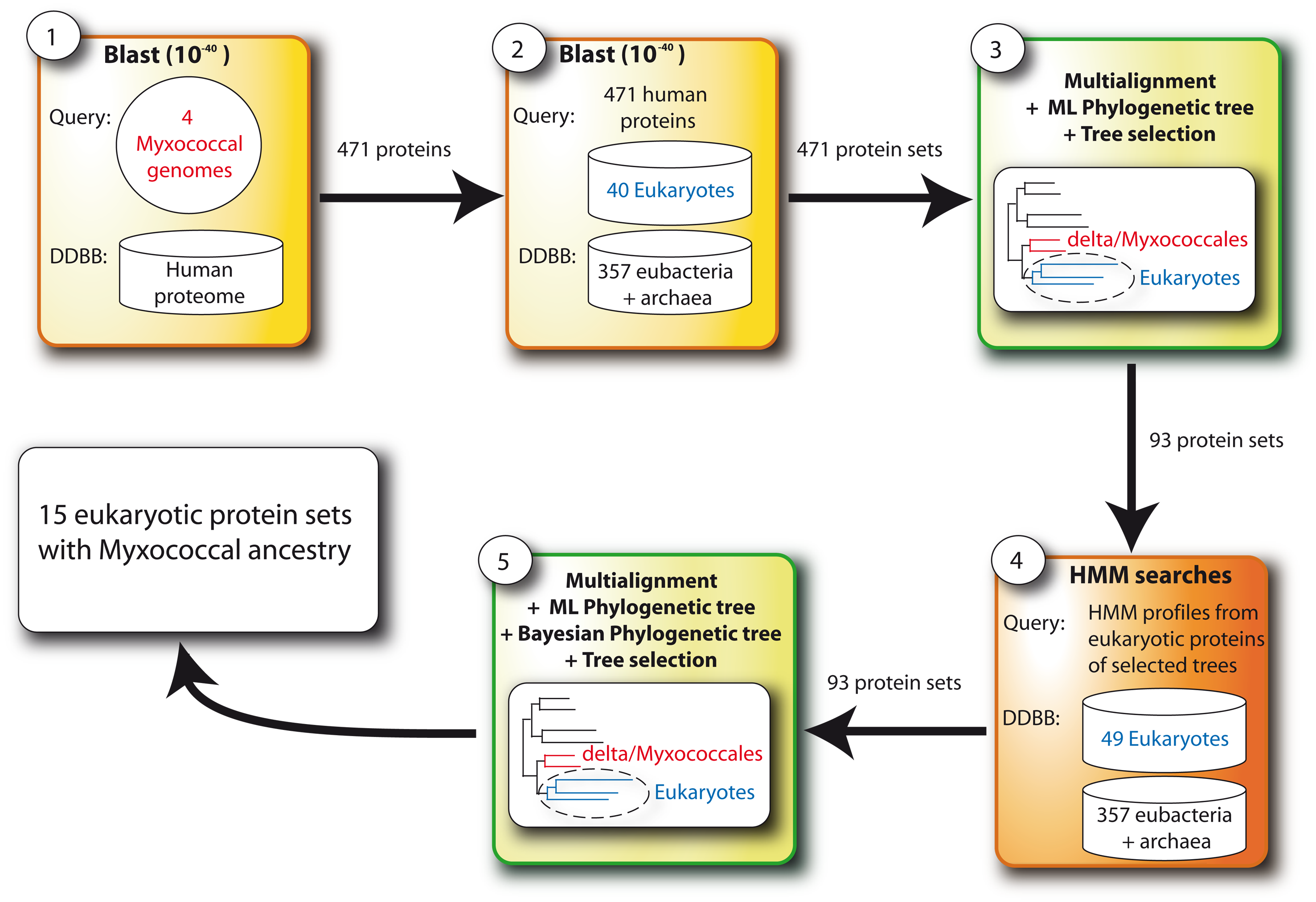

Supplement: Figure S1 — Schematic diagram of the automatic pipeline followed to identify eukaryotic proteins with a predicted myxococcal origin. (TIF) [file pone.0021989.s001.tif]

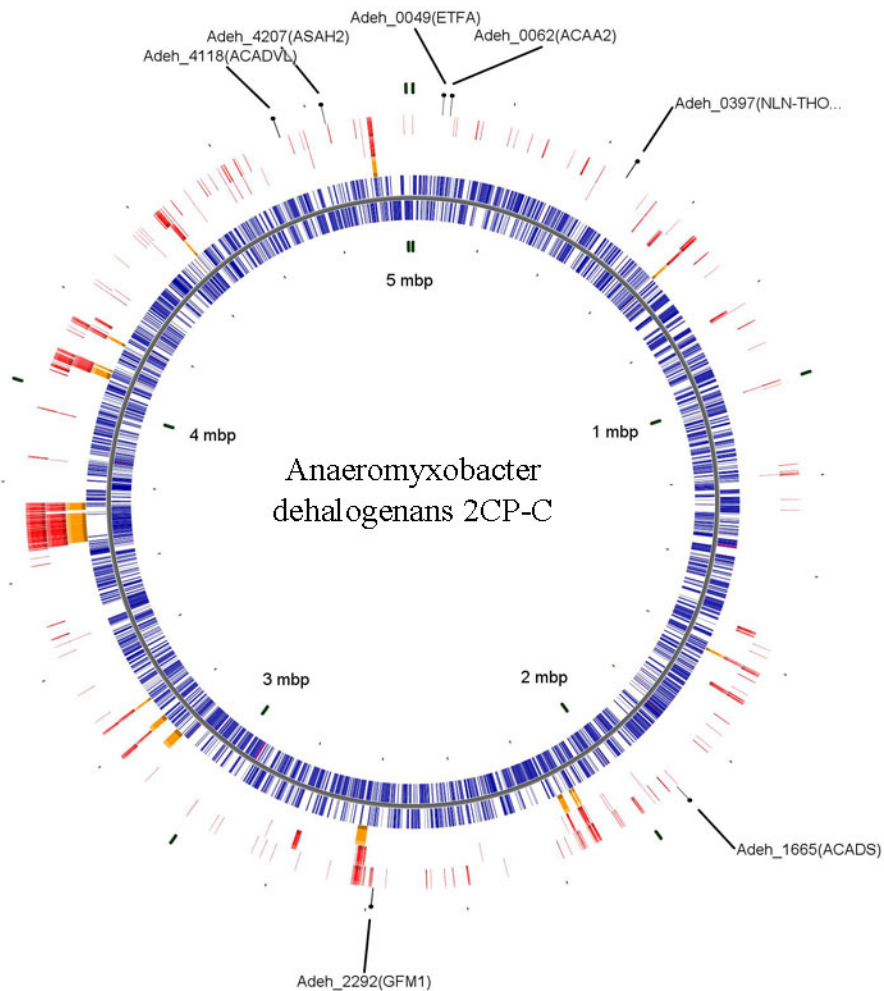

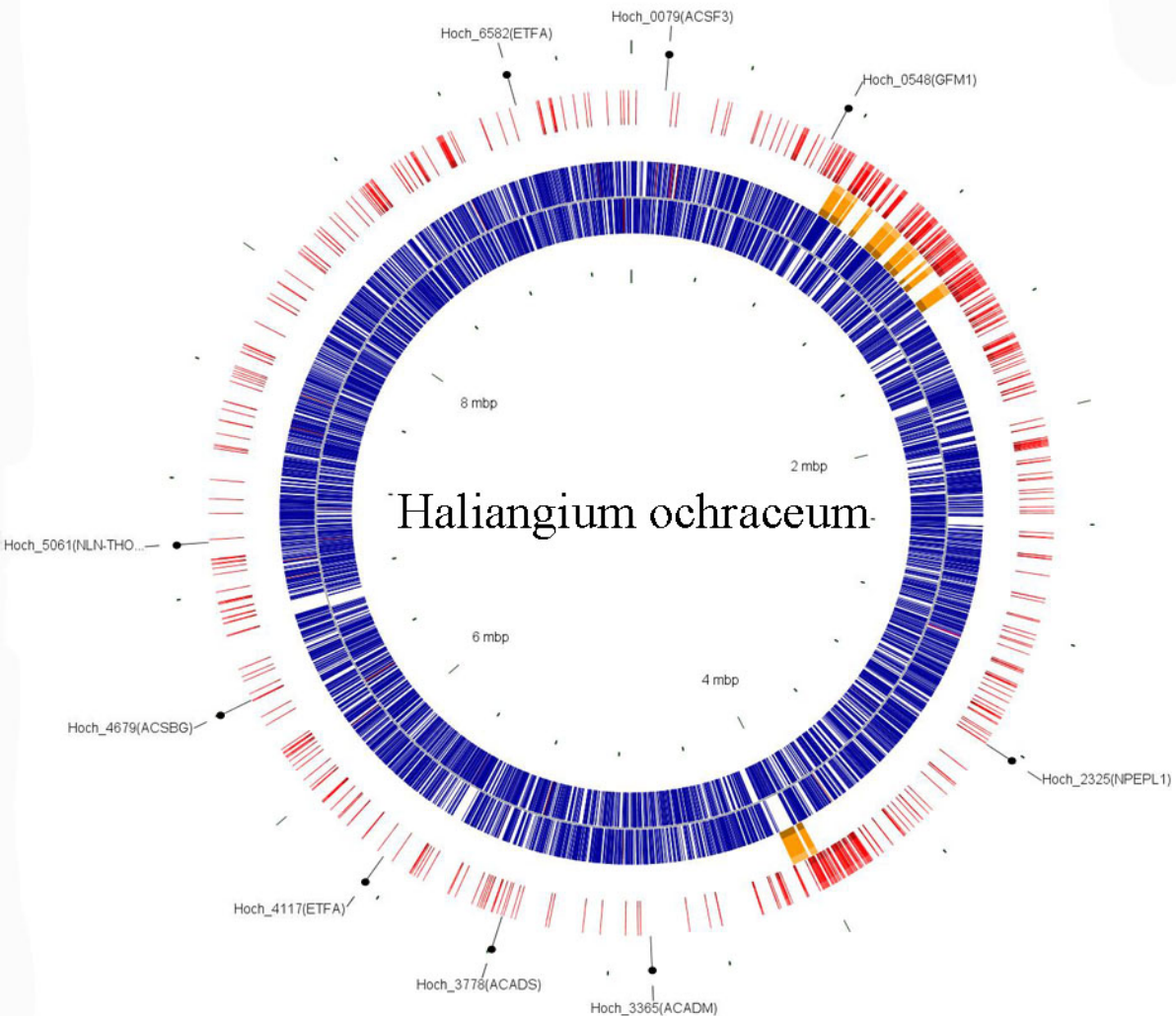

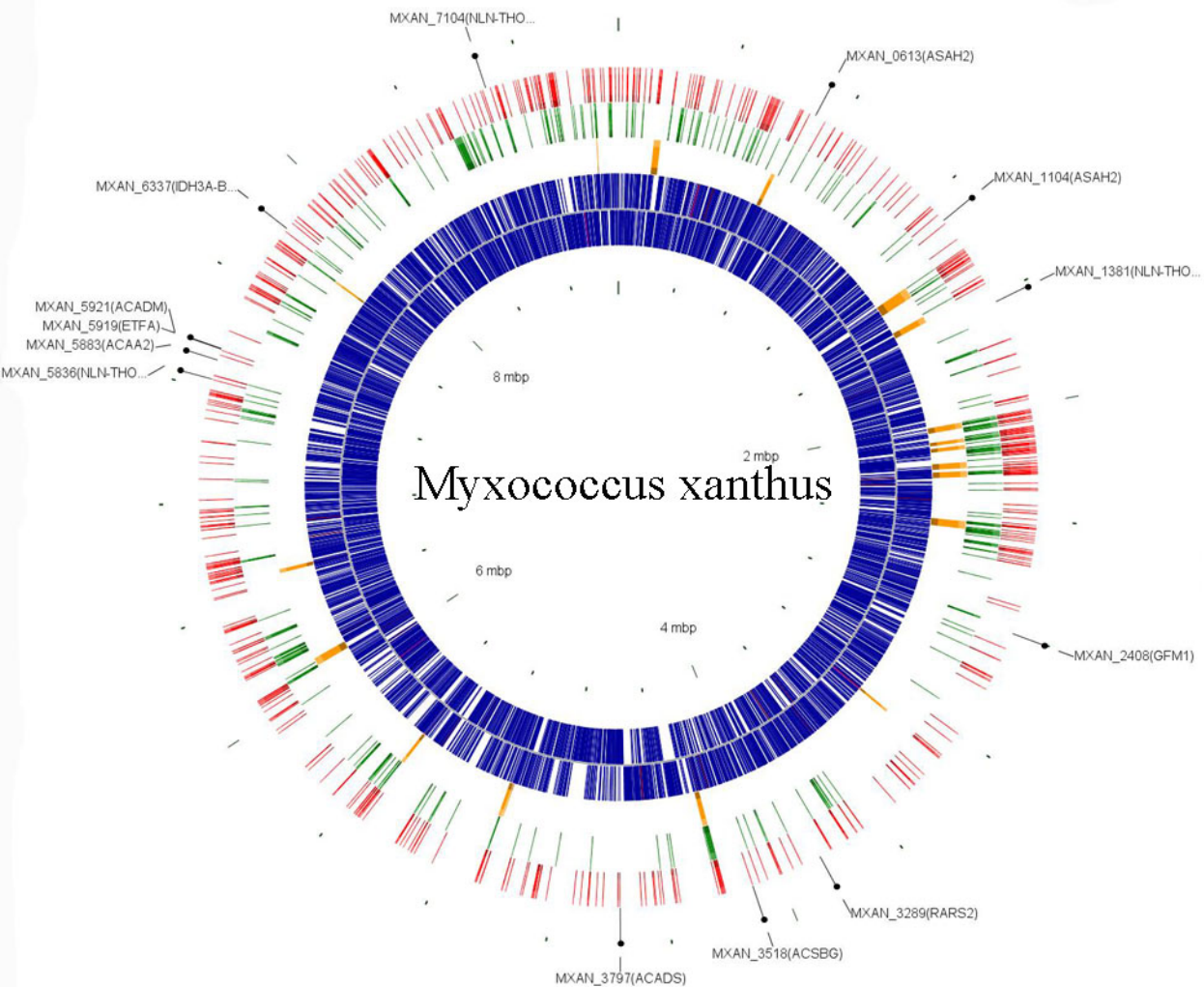

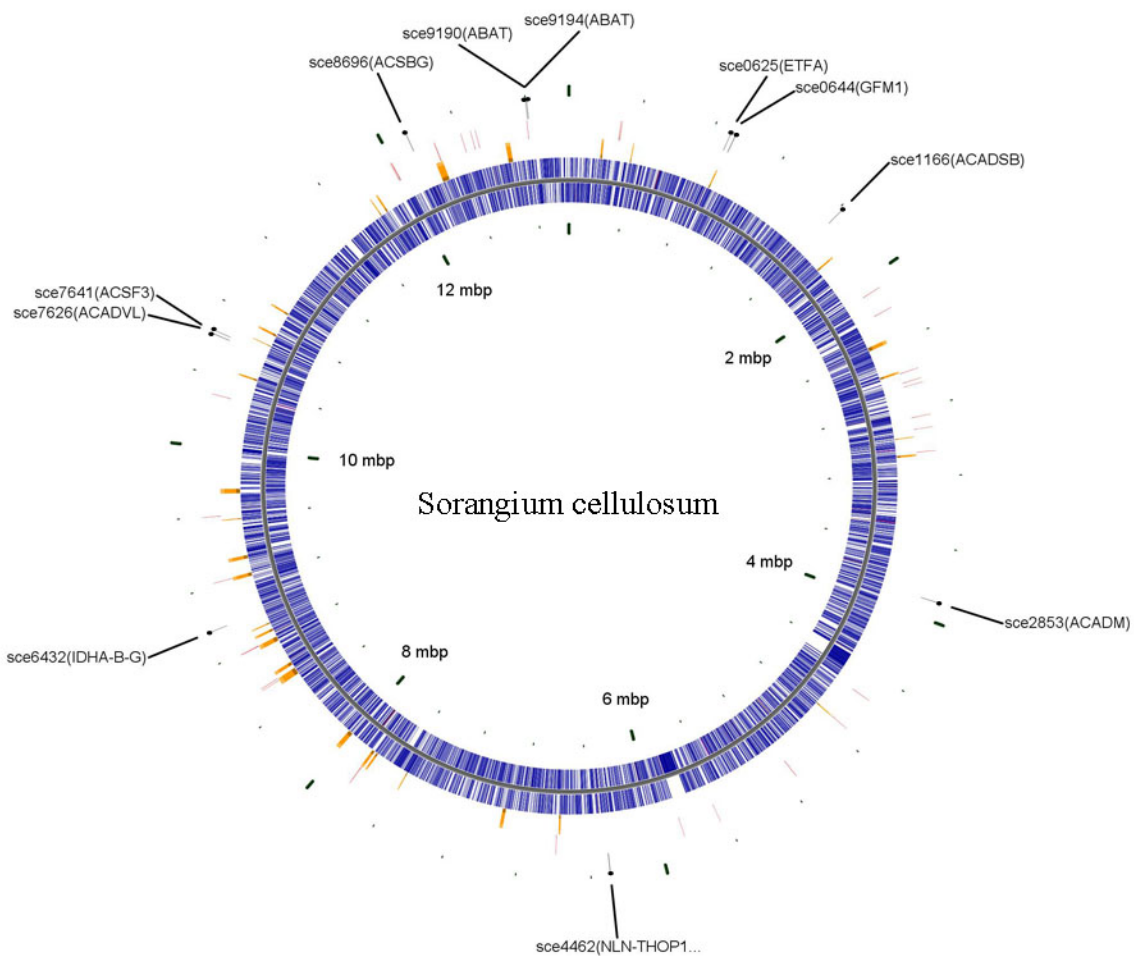

Supplement: Figure S2 — Circular genome maps of A. dehalogenans, H. ochraceum, M xanthus and S. cellulosum, showing their respective ORFs. In the outermost layer of the circle, the proposed myxoccocal ancestor genes are indicated as black stripes. The abbreviated names of the corresponding eukaryotic proteins are indicated in parentheses. Putative gene acquisitions via lateral gene transfer are displayed with three different methods: a) alien genes via the Karlin method in red, b) lateral gene transfers from HGT-DB in green, and c) genomic islands using the Island Viewer server in yellow. (PDF) [file pone.0021989.s002.pdf]

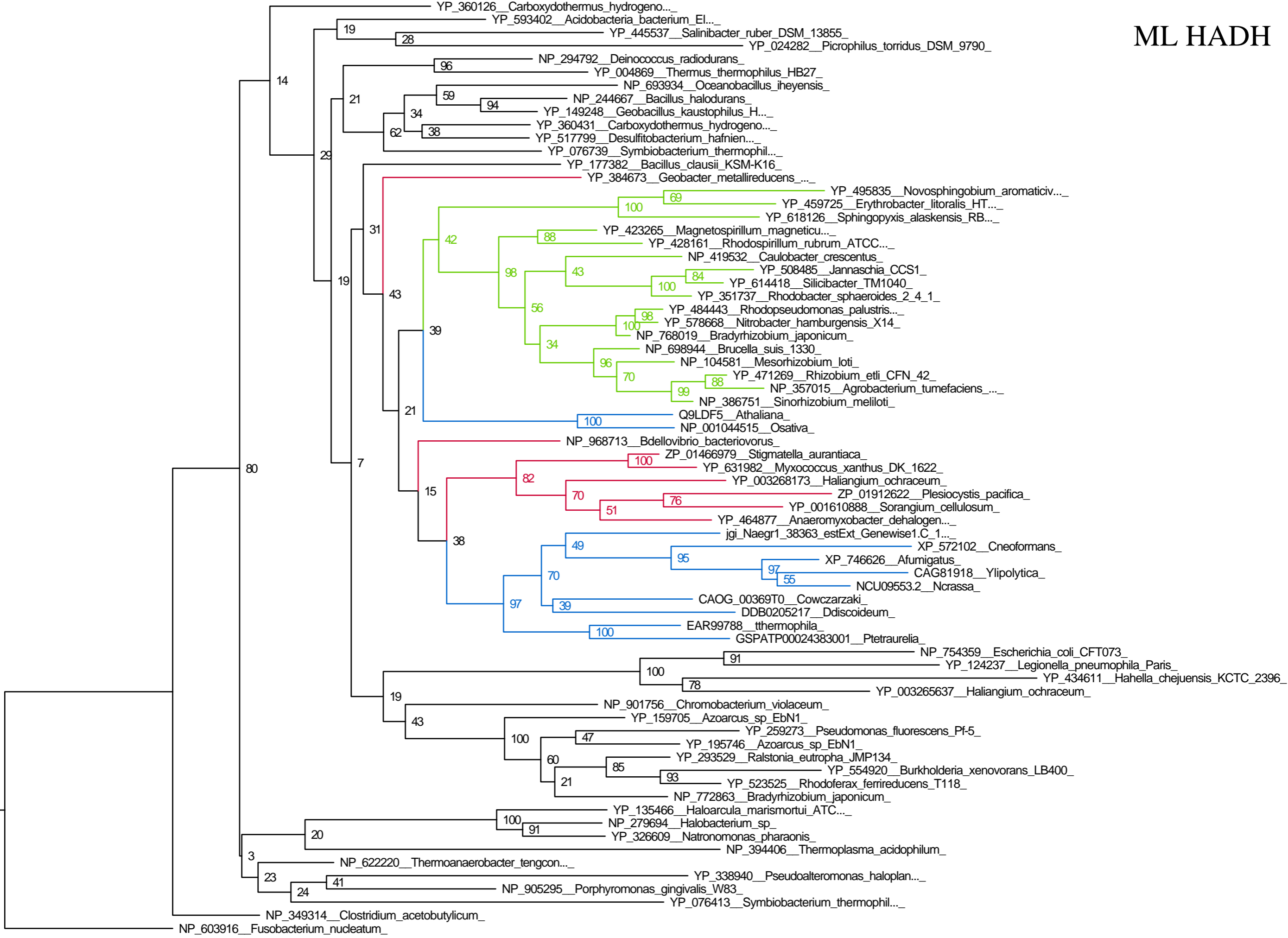

Bayesian HADH

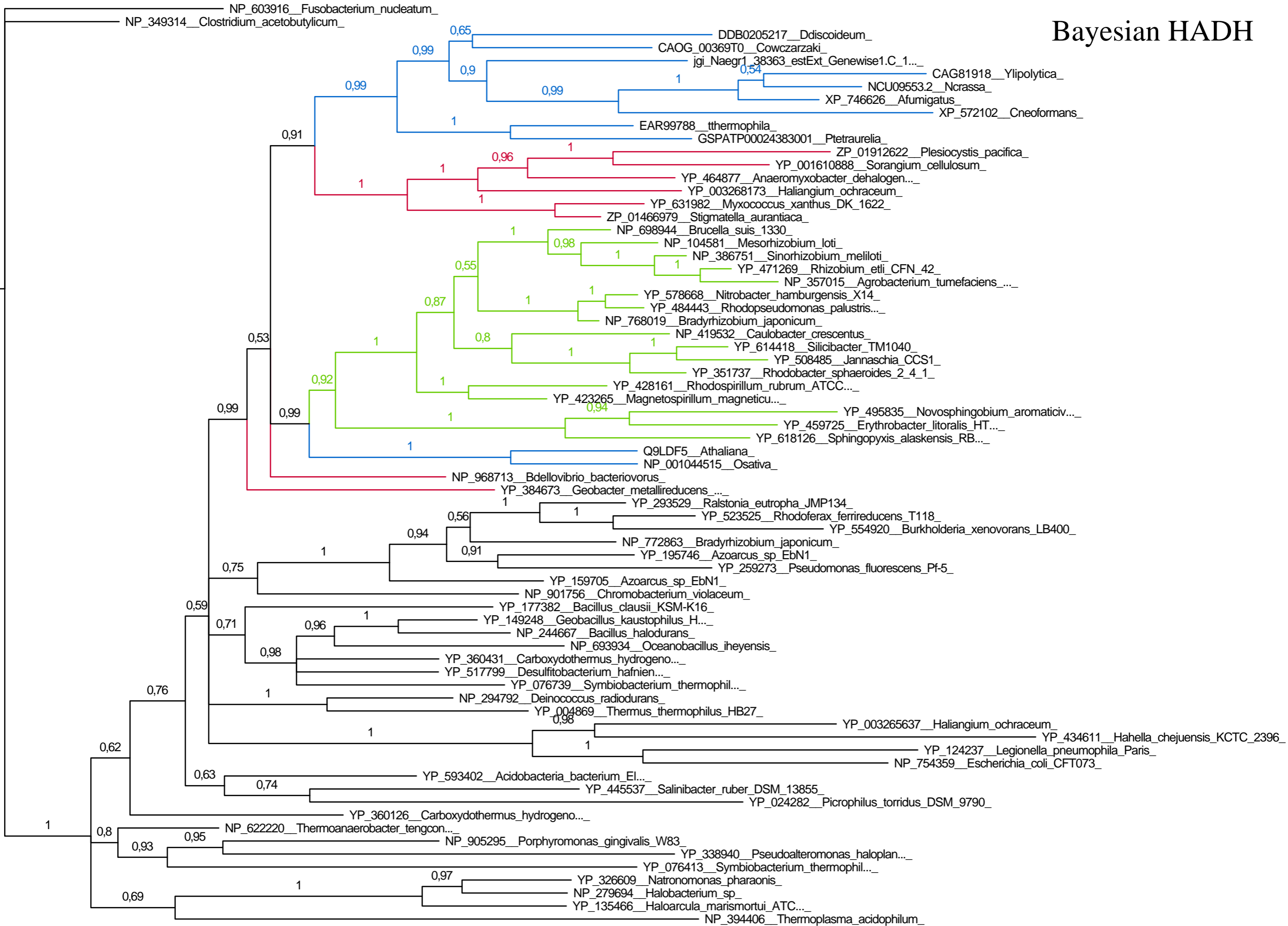

Supplement: Figure S3 — ML and Bayesian phylogenetic tree of a monofunctional hydroxyacyl-CoA dehydrogenase (HADH) protein. Eukaryotic, myxococcal/δ-proteobacterial and α-proteobacterial taxa are highlighted in blue, red and green, respectively. (PDF) [file pone.0021989.s003.pdf]
