# Supplementary material for: Phylogenomic Evidence for a Myxococcal Contribution to the Mitochondrial Fatty Acid Beta-Oxidation
Source: PLoS One. 2011 Jul 7;6(7):e21989. doi: 10.1371/journal.pone.0021989 (PMC3131387; doi:10.1371/journal.pone.0021989)

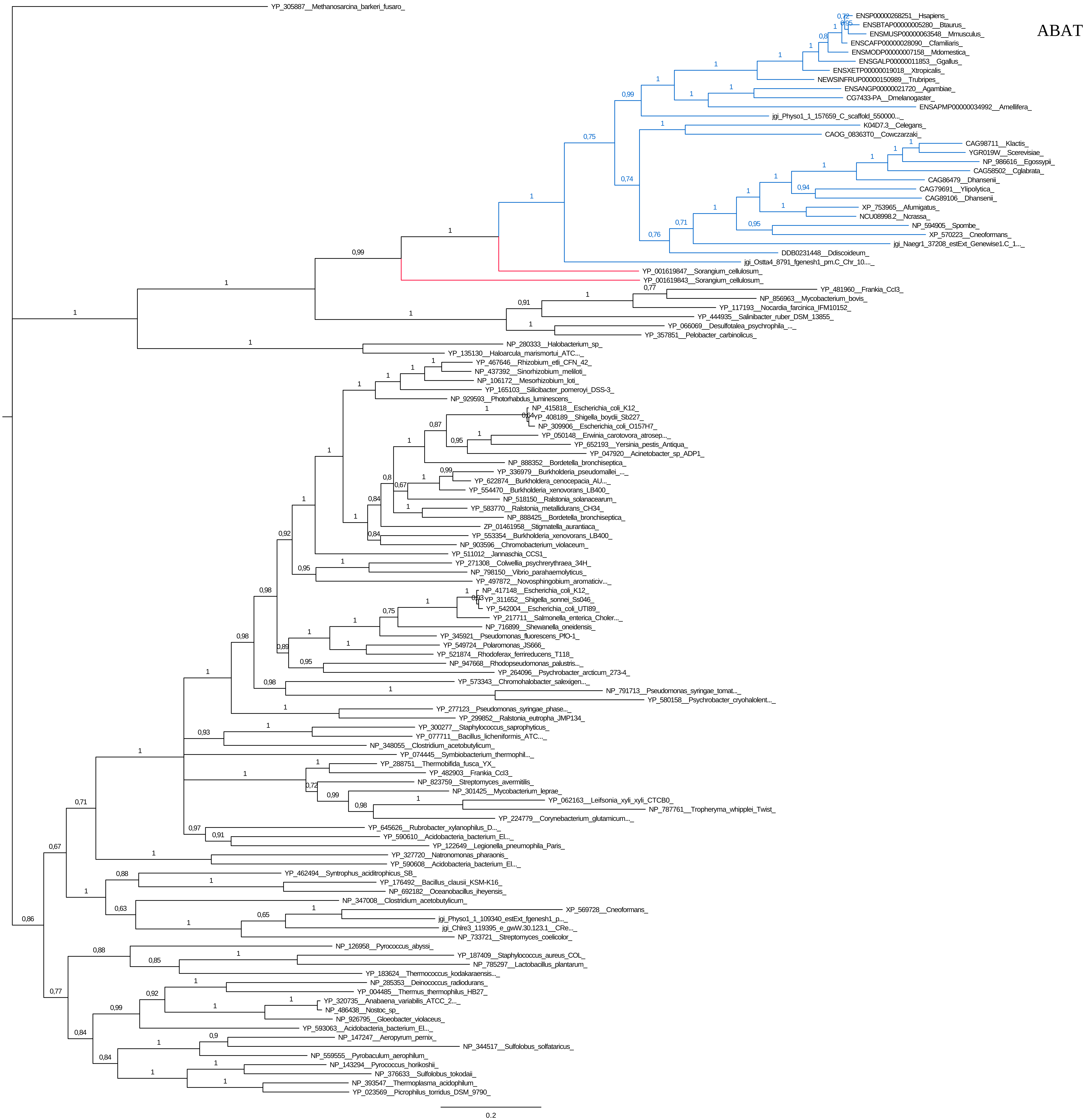
ABAT
YP_305887__Methanosarcina_barkeri_fusaro_
ENSP00000268251__Hsapiens_
ENSBTAP00000005280__Btaurus_
ENSMUSP00000063548__Mmusculus_
ENSCAFP00000028090__Cfamiliaris_
ENSMODP00000007158__Mdomestica_
ENSGALP00000011853__Ggallus_
ENSXETP00000019018__Xtropicalis_
NEWSINFRUP00000150989__Trubripes_
ENSANGP00000021720__Agambiae_
CG7433-PA__Dmelanogaster_
ENSAPMP00000034992__Amellifera_
jgi_Physo1_1_157659_C_scaffold_550000..._
K04D7.3__Celegans_
CAOG_08363T0__Cowczarzaki_
CAG98711__Klactis_
YGR019W__Scerevisiae_
NP_986616__Egossypii_
CAG58502__Cglabrata_
CAG86479__Dhansenii_
CAG79691__Ylipolytica_
CAG89106__Dhansenii_
XP_753965__Afumigatus_
NCU08998.2__Ncrassa_
NP_594905__Spombe_
XP_570223__Cneoformans_
jgi_Naegr1_37208_estExt_Genewise1.C_1..._
DDB0231448__Ddiscoideum_
jgi_Ostta4_8791_fgenesh1_pm.C_Chr_10...._
YP_001619847__Sorangium_cellulosum_
YP_001619843__Sorangium_cellulosum_
YP_481960__Frankia_CcI3_
NP_856963__Mycobacterium_bovis_
YP_117193__Nocardia_farcinica_IFM10152_
YP_444935__Salinibacter_ruber_DSM_13855_
YP_066069__Desulfotalea_psychrophila_..._
YP_357851__Pelobacter_carbinolicus_
NP_280333__Halobacterium_sp_
YP_135130__Haloarcula_marismortui_ATC..._
YP_467646__Rhizobium_etli_CFN_42_
NP_437392__Sinorhizobium_meliloti_
NP_106172__Mesorhizobium_loti_
YP_165103__Silicibacter_pomeroyi_DSS-3_
NP_929593__Photorhabdus_luminescens_
NP_415818__Escherichia_coli_K12_
YP_408189__Shigella_boydii_Sb227_
NP_309906__Escherichia_coli_O157H7_
YP_050148__Erwinia_carotovora_atrosep..._
YP_652193__Yersinia_pestis_Antiqua_
YP_047920__Acinetobacter_sp_ADP1_
NP_888352__Bordetella_bronchiseptica_
YP_336979__Burkholderia_pseudomallei_..._
YP_622874__Burkholdera_cenocepacia_AU..._
YP_554470__Burkholderia_xenovorans_LB400_
NP_518150__Ralstonia_solanacearum_
YP_583770__Ralstonia_metallidurans_CH34_
NP_888425__Bordetella_bronchiseptica_
ZP_01461958__Stigmatella_aurantiaca_
YP_553354__Burkholderia_xenovorans_LB400_
NP_903596__Chromobacterium_violaceum_
YP_511012__Jannaschia_CCS1_
YP_271308__Colwellia_psychrerythraea_34H_
NP_798150__Vibrio_parahaemolyticus_
YP_497872__Novosphingobium_aromaticiv..._
NP_417148__Escherichia_coli_K12_
YP_311652__Shigella_sonnei_Ss046_
YP_542004__Escherichia_coli_UTI89_
YP_217711__Salmonella_enterica_Choler..._
NP_716899__Shewanella_oneidensis_
YP_345921__Pseudomonas_fluorescens_PfO-1_
YP_549724__Polaromonas_JS666_
YP_521874__Rhodoferax_ferrireducens_T118_
NP_947668__Rhodopseudomonas_palustris..._
YP_264096__Psychrobacter_arcticum_273-4_
YP_573343__Chromohalobacter_salexigen..._
NP_791713__Pseudomonas_syringae_tomat..._
YP_580158__Psychrobacter_cryohalolent..._
YP_277123__Pseudomonas_syringae_phase..._
YP_299852__Ralstonia_eutropha_JMP134_
YP_300277__Staphylococcus_saprophyticus_
YP_077711__Bacillus_licheniformis_ATC..._
NP_348055__Clostridium_acetobutylicum_
YP_074445__Symbiobacterium_thermophil..._
YP_288751__Thermobifida_fusca_YX_
YP_482903__Frankia_CcI3_
NP_823759__Streptomyces_avermitilis_
NP_301425__Mycobacterium_leprae_
YP_062163__Leifsonia_xyli_xyli_CTCB0_
NP_787761__Tropheryma_whipplei_Twist_
YP_224779__Corynebacterium_glutamicum..._
YP_645626__Rubrobacter_xylanophilus_D..._
YP_590610__Acidobacteria_bacterium_El..._
YP_122649__Legionella_pneumophila_Paris_
YP_327720__Natronomonas_pharaonis_
YP_590608__Acidobacteria_bacterium_El..._
YP_462494__Syntrophus_aciditrophicus_SB_
YP_176492__Bacillus_clausii_KSM-K16_
NP_692182__Oceanobacillus_iheyensis_
NP_347008__Clostridium_acetobutylicum_
XP_569728__Cneoformans_
jgi_Physo1_1_109340_estExt_fgenesh1_p..._
jgi_Chlre3_119395_e_gwW.30.123.1__CRe..._
NP_733721__Streptomyces_coelicolor_
NP_126958__Pyrococcus_abyssi_
YP_187409__Staphylococcus_aureus_COL_
NP_785297__Lactobacillus_plantarum_
YP_183624__Thermococcus_kodakaraensis..._
NP_285353__Deinococcus_radiodurans_
YP_004485__Thermus_thermophilus_HB27_
YP_320735__Anabaena_variabilis_ATCC_2..._
NP_486438__Nostoc_sp_
NP_926795__Gloeobacter_violaceus_
YP_593063__Acidobacteria_bacterium_El..._
NP_147247__Aeropyrum_pernix_
NP_344517__Sulfolobus_solfataricus_
NP_559555__Pyrobaculum_aerophilum_
NP_143294__Pyrococcus_horikoshii_
NP_376633__Sulfolobus_tokodaii_
NP_393547__Thermoplasma_acidophilum_
YP_023569__Picrophilus_torridus_DSM_9790_
0.2

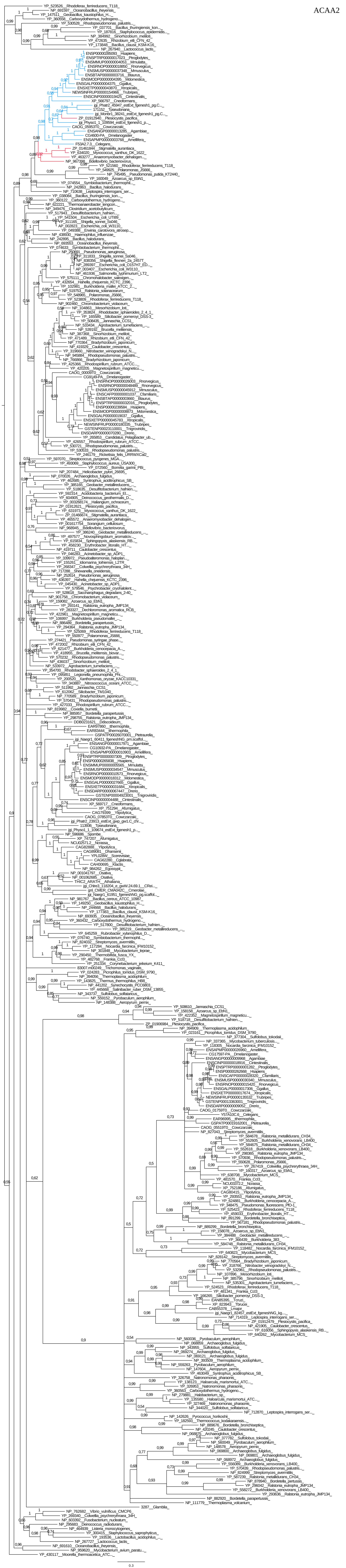
ACAA2
0.3

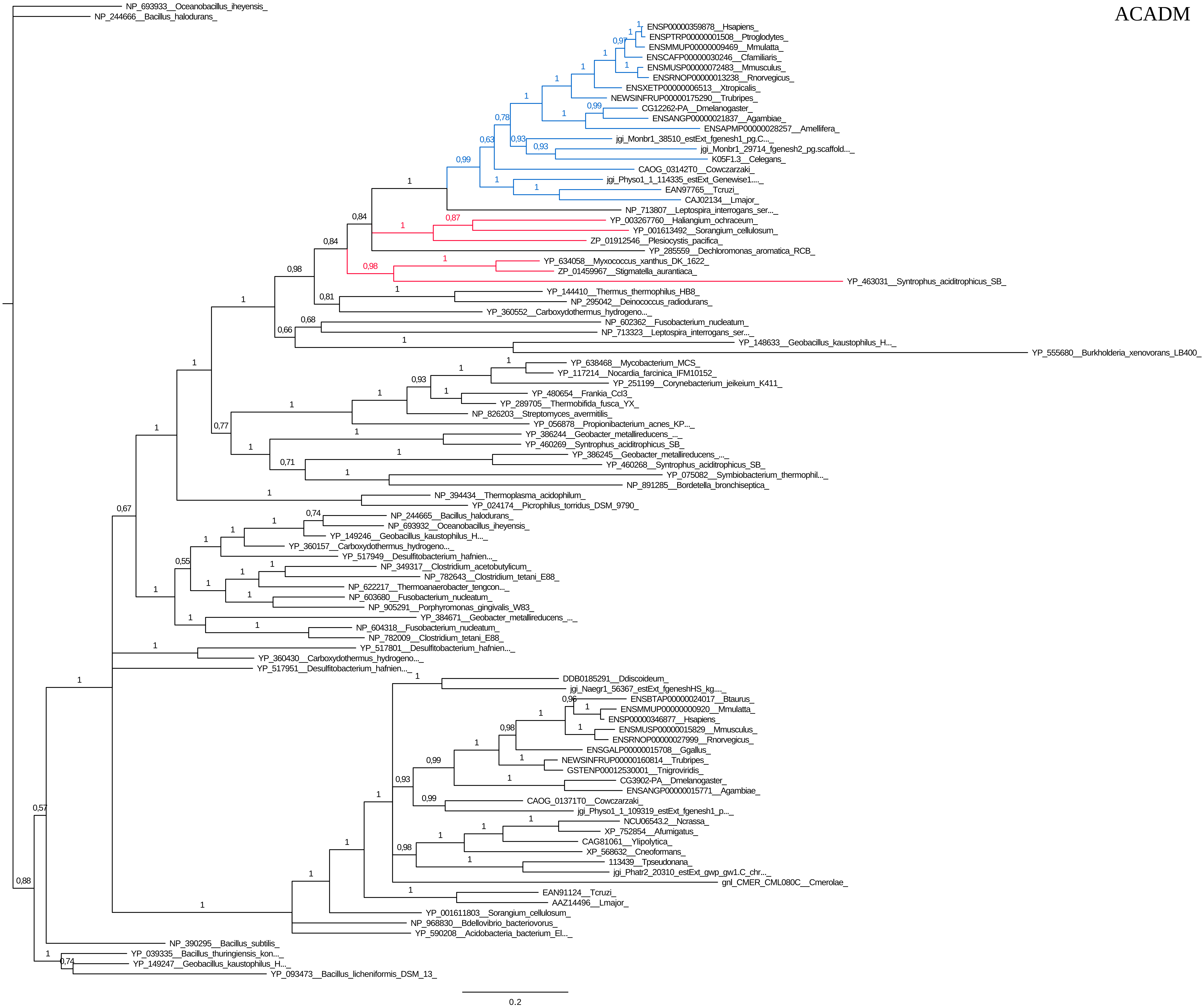
ACADM
NP_693933__Oceanobacillus_iheyensis_
NP_244666__Bacillus_halodurans_
ENSP00000359878__Hsapiens_
ENSPTRP00000001508__Ptroglodytes_
ENSMMUP00000009469__Mmulatta_
ENSCAFP00000030246__Cfamiliaris_
ENSMUSP00000072483__Mmusculus_
ENSRNOP00000013238__Rnorvegicus_
ENSXETP00000006513__Xtropicalis_
NEWSINFRUP00000175290__Trubripes_
CG12262-PA__Dmelanogaster_
ENSANGP00000021837__Agambiae_
ENSAPMP00000028257__Amellifera_
jgi_Monbr1_38510_estExt_fgenesh1_pg.C..._
jgi_Monbr1_29714_fgenesh2_pg.scaffold..._
K05F1.3__Celegans_
CAOG_03142T0__Cowczarzaki_
jgi_Physo1_1_114335_estExt_Genewise1...._
EAN97765__Tcruzi_
CAJ02134__Lmajor_
NP_713807__Leptospira_interrogans_ser..._
YP_003267760__Haliangium_ochraceum_
YP_001613492__Sorangium_cellulosum_
ZP_01912546__Plesiocystis_pacifica_
YP_285559__Dechloromonas_aromatica_RCB_
YP_634058__Myxococcus_xanthus_DK_1622_
ZP_01459967__Stigmatella_aurantiaca_
YP_463031__Syntrophus_aciditrophicus_SB_
YP_144410__Thermus_thermophilus_HB8_
NP_295042__Deinococcus_radiodurans_
YP_360552__Carboxydothermus_hydrogeno..._
NP_602362__Fusobacterium_nucleatum_
NP_713323__Leptospira_interrogans_ser..._
YP_148633__Geobacillus_kaustophilus_H..._
YP_555680__Burkholderia_xenovorans_LB400_
YP_638468__Mycobacterium_MCS_
YP_117214__Nocardia_farcinica_IFM10152_
YP_251199__Corynebacterium_jeikeium_K411_
YP_480654__Frankia_CcI3_
YP_289705__Thermobifida_fusca_YX_
NP_826203__Streptomyces_avermitilis_
YP_056878__Propionibacterium_acnes_KP..._
YP_386244__Geobacter_metallireducens_..._
YP_460269__Syntrophus_aciditrophicus_SB_
YP_386245__Geobacter_metallireducens_..._
YP_460268__Syntrophus_aciditrophicus_SB_
YP_075082__Symbiobacterium_thermophil..._
NP_891285__Bordetella_bronchiseptica_
NP_394434__Thermoplasma_acidophilum_
YP_024174__Picrophilus_torridus_DSM_9790_
NP_244665__Bacillus_halodurans_
NP_693932__Oceanobacillus_iheyensis_
YP_149246__Geobacillus_kaustophilus_H..._
YP_360157__Carboxydothermus_hydrogeno..._
YP_517949__Desulfitobacterium_hafnien..._
NP_349317__Clostridium_acetobutylicum_
NP_782643__Clostridium_tetani_E88_
NP_622217__Thermoanaerobacter_tengcon..._
NP_603680__Fusobacterium_nucleatum_
NP_905291__Porphyromonas_gingivalis_W83_
YP_384671__Geobacter_metallireducens_..._
NP_604318__Fusobacterium_nucleatum_
NP_782009__Clostridium_tetani_E88_
YP_517801__Desulfitobacterium_hafnien..._
YP_360430__Carboxydothermus_hydrogeno..._
YP_517951__Desulfitobacterium_hafnien..._
DDB0185291__Ddiscoideum_
jgi_Naegr1_56367_estExt_fgeneshHS_kg...._
ENSBTAP00000024017__Btaurus_
ENSMMUP00000000920__Mmulatta_
ENSP00000346877__Hsapiens_
ENSMUSP00000015829__Mmusculus_
ENSRNOP00000027999__Rnorvegicus_
ENSGALP00000015708__Ggallus_
NEWSINFRUP00000160814__Trubripes_
GSTENP00012530001__Tnigroviridis_
CG3902-PA__Dmelanogaster_
ENSANGP00000015771__Agambiae_
CAOG_01371T0__Cowczarzaki_
jgi_Physo1_1_109319_estExt_fgenesh1_p..._
NCU06543.2__Ncrassa_
XP_752854__Afumigatus_
CAG81061__Ylipolytica_
XP_568632__Cneoformans_
113439__Tpseudonana_
jgi_Phatr2_20310_estExt_gwp_gw1.C_chr..._
gnl_CMER_CML080C__Cmerolae_
EAN91124__Tcruzi_
AAZ14496__Lmajor_
YP_001611803__Sorangium_cellulosum_
NP_968830__Bdellovibrio_bacteriovorus_
YP_590208__Acidobacteria_bacterium_El..._
NP_390295__Bacillus_subtilis_
YP_039335__Bacillus_thuringiensis_kon..._
YP_149247__Geobacillus_kaustophilus_H..._
YP_093473__Bacillus_licheniformis_DSM_13_
0.2

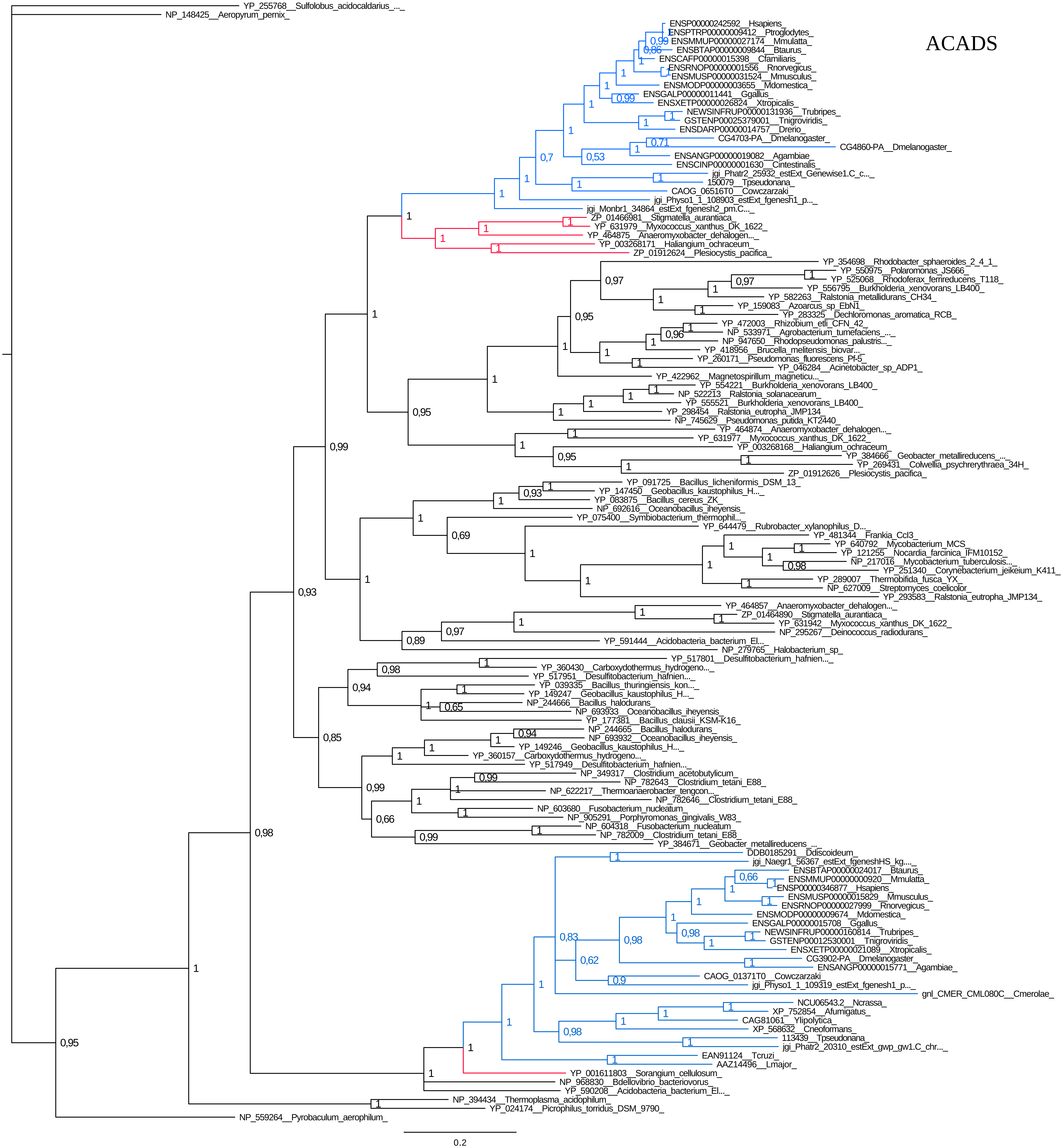
ACADS
0.2

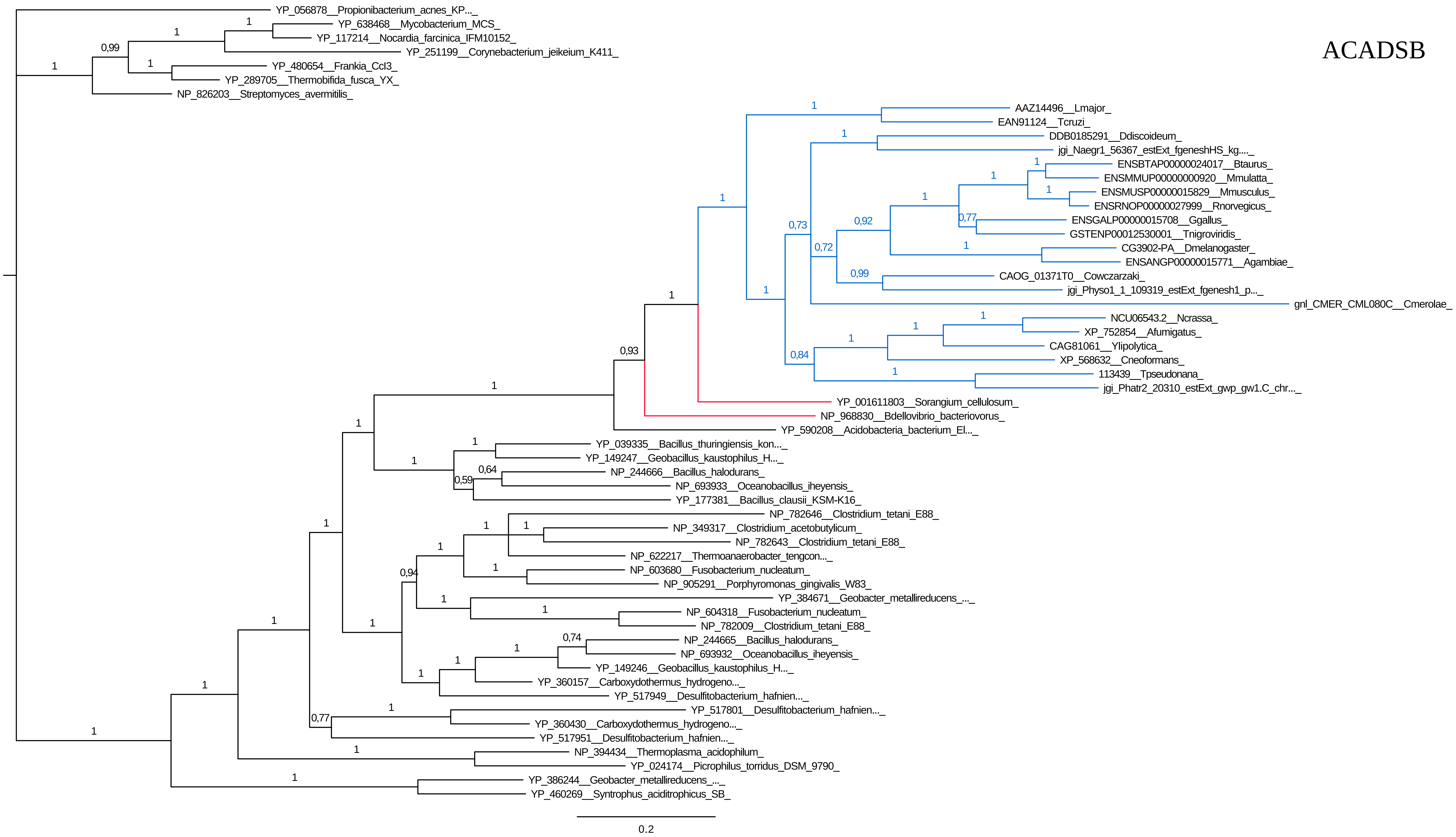
ACADSB
YP_056878__Propionibacterium_acnes_KP..._
YP_638468__Mycobacterium_MCS_
YP_117214__Nocardia_farcinica_IFM10152_
YP_251199__Corynebacterium_jeikeium_K411_
YP_480654__Frankia_CcI3_
YP_289705__Thermobifida_fusca_YX_
NP_826203__Streptomyces_avermitilis_
AAZ14496__Lmajor_
EAN91124__Tcruzi_
DDB0185291__Ddiscoideum_
jgi_Naegr1_56367_estExt_fgeneshHS_kg...._
ENSBTAP00000024017__Btaurus_
ENSMMUP00000000920__Mmulatta_
ENSMUSP00000015829__Mmusculus_
ENSRNOP00000027999__Rnorvegicus_
ENSGALP00000015708__Ggallus_
GSTENP00012530001__Tnigroviridis_
CG3902-PA__Dmelanogaster_
ENSANGP00000015771__Agambiae_
CAOG_01371T0__Cowczarzaki_
jgi_Physo1_1_109319_estExt_fgenesh1_p..._
gnl_CMER_CML080C__Cmerolae_
NCU06543.2__Ncrassa_
XP_752854__Afumigatus_
CAG81061__Ylipolytica_
XP_568632__Cneoformans_
113439__Tpseudonana_
jgi_Phatr2_20310_estExt_gwp_gw1.C_chr..._
YP_001611803__Sorangium_cellulosum_
NP_968830__Bdellovibrio_bacteriovorus_
YP_590208__Acidobacteria_bacterium_El..._
YP_039335__Bacillus_thuringiensis_kon..._
YP_149247__Geobacillus_kaustophilus_H..._
NP_244666__Bacillus_halodurans_
NP_693933__Oceanobacillus_iheyensis_
YP_177381__Bacillus_clausii_KSM-K16_
NP_782646__Clostridium_tetani_E88_
NP_349317__Clostridium_acetobutylicum_
NP_782643__Clostridium_tetani_E88_
NP_622217__Thermoanaerobacter_tengcon..._
NP_603680__Fusobacterium_nucleatum_
NP_905291__Porphyromonas_gingivalis_W83_
YP_384671__Geobacter_metallireducens_..._
NP_604318__Fusobacterium_nucleatum_
NP_782009__Clostridium_tetani_E88_
NP_244665__Bacillus_halodurans_
NP_693932__Oceanobacillus_iheyensis_
YP_149246__Geobacillus_kaustophilus_H..._
YP_360157__Carboxydothermus_hydrogeno..._
YP_517949__Desulfitobacterium_hafnien..._
YP_517801__Desulfitobacterium_hafnien..._
YP_360430__Carboxydothermus_hydrogeno..._
YP_517951__Desulfitobacterium_hafnien..._
NP_394434__Thermoplasma_acidophilum_
YP_024174__Picrophilus_torridus_DSM_9790_
YP_386244__Geobacter_metallireducens_..._
YP_460269__Syntrophus_aciditrophicus_SB_
0.2

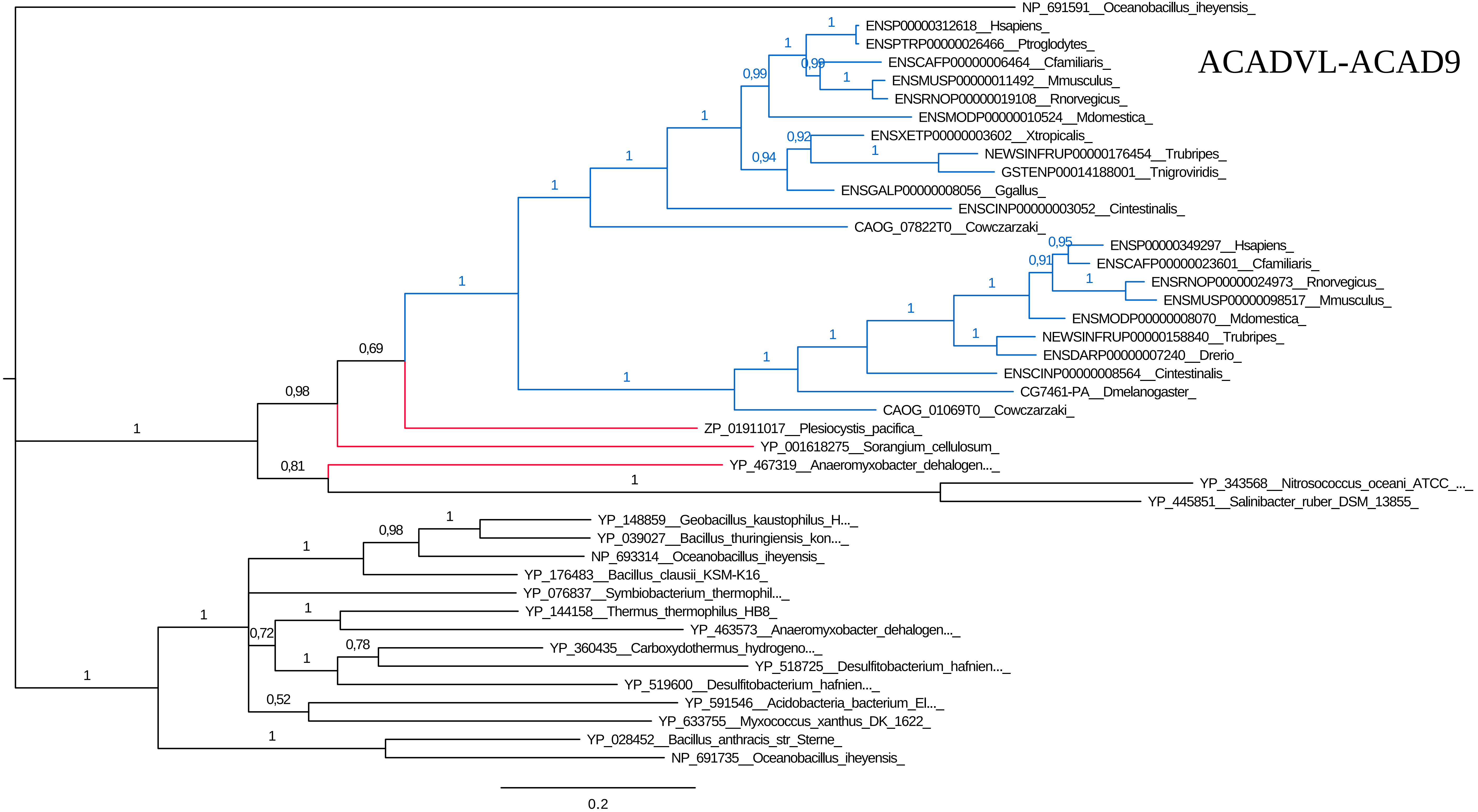
ACADVL-ACAD9
NP_691591__Oceanobacillus_iheyensis_
ENSP00000312618__Hsapiens_
ENSPTRP00000026466__Ptroglodytes_
ENSCAFP00000006464__Cfamiliaris_
ENSMUSP00000011492__Mmusculus_
ENSRNOP00000019108__Rnorvegicus_
ENSMODP00000010524__Mdomestica_
ENSXETP00000003602__Xtropicalis_
NEWSINFRUP00000176454__Trubripes_
GSTENP00014188001__Tnigroviridis_
ENSGALP00000008056__Ggallus_
ENSCINP00000003052__Cintestinalis_
CAOG_07822T0__Cowczarzaki_
ENSP00000349297__Hsapiens_
ENSCAFP00000023601__Cfamiliaris_
ENSRNOP00000024973__Rnorvegicus_
ENSMUSP00000098517__Mmusculus_
ENSMODP00000008070__Mdomestica_
NEWSINFRUP00000158840__Trubripes_
ENSDARP00000007240__Drerio_
ENSCINP00000008564__Cintestinalis_
CG7461-PA__Dmelanogaster_
CAOG_01069T0__Cowczarzaki_
ZP_01911017__Plesiocystis_pacifica_
YP_001618275__Sorangium_cellulosum_
YP_467319__Anaeromyxobacter_dehalogen..._
YP_343568__Nitrosococcus_oceani_ATCC_..._
YP_445851__Salinibacter_ruber_DSM_13855_
YP_148859__Geobacillus_kaustophilus_H..._
YP_039027__Bacillus_thuringiensis_kon..._
NP_693314__Oceanobacillus_iheyensis_
YP_176483__Bacillus_clausii_KSM-K16_
YP_076837__Symbiobacterium_thermophil..._
YP_144158__Thermus_thermophilus_HB8_
YP_463573__Anaeromyxobacter_dehalogen..._
YP_360435__Carboxydothermus_hydrogeno..._
YP_518725__Desulfitobacterium_hafnien..._
YP_519600__Desulfitobacterium_hafnien..._
YP_591546__Acidobacteria_bacterium_El..._
YP_633755__Myxococcus_xanthus_DK_1622_
YP_028452__Bacillus_anthracis_str_Sterne_
NP_691735__Oceanobacillus_iheyensis_
0.2

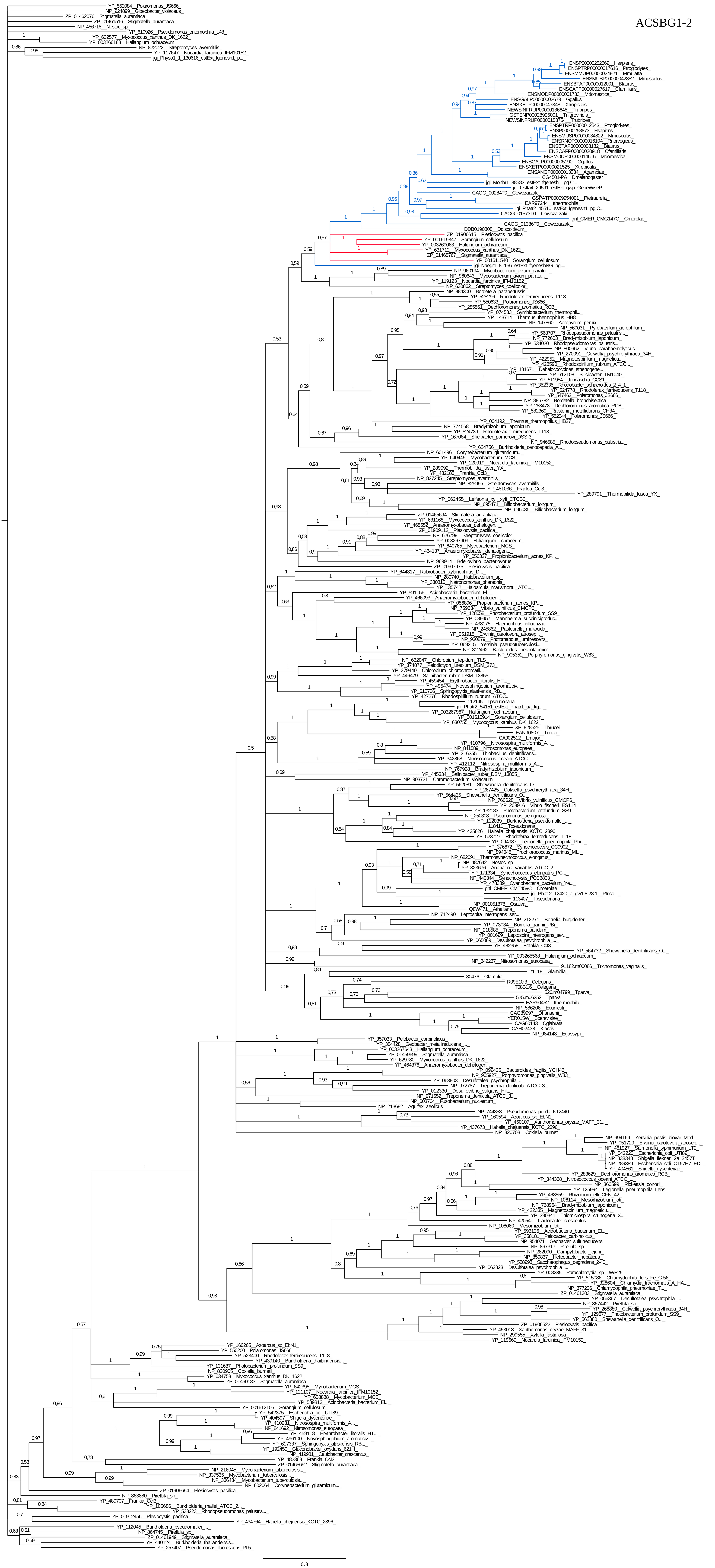
ACSBG1-2
0.3

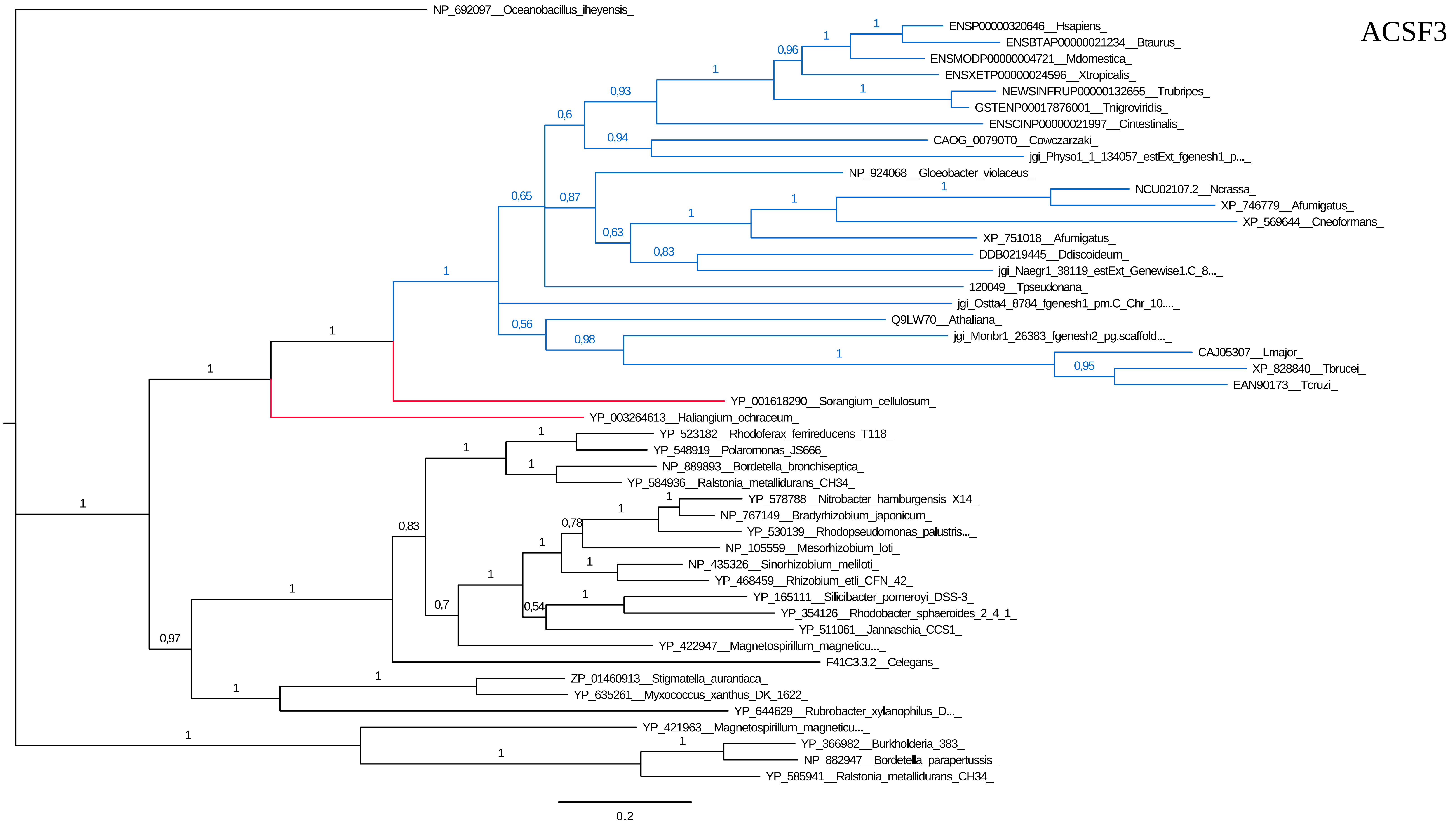

ACSF3
NP_692097__Oceanobacillus_iheyensis_
ENSP00000320646__Hsapiens_
ENSBTAP00000021234__Btaurus_
ENSMODP00000004721__Mdomestica_
ENSXETP00000024596__Xtropicalis_
NEWSINFRUP00000132655__Trubripes_
GSTENP00017876001__Tnigroviridis_
ENSCINP00000021997__Cintestinalis_
CAOG_00790T0__Cowczarzaki_
jgi_Physo1_1_134057_estExt_fgenesh1_p..._
NP_924068__Gloeobacter_violaceus_
NCU02107.2__Ncrassa_
XP_746779__Afumigatus_
XP_569644__Cneoformans_
XP_751018__Afumigatus_
DDB0219445__Ddiscoideum_
jgi_Naegr1_38119_estExt_Genewise1.C_8..._
120049__Tpseudonana_
jgi_Ostta4_8784_fgenesh1_pm.C_Chr_10...._
Q9LW70__Athaliana_
jgi_Monbr1_26383_fgenesh2_pg.scaffold..._
CAJ05307__Lmajor_
XP_828840__Tbrucei_
EAN90173__Tcruzi_
YP_001618290__Sorangium_cellulosum_
YP_003264613__Haliangium_ochraceum_
YP_523182__Rhodoferax_ferrireducens_T118_
YP_548919__Polaromonas_JS666_
NP_889893__Bordetella_bronchiseptica_
YP_584936__Ralstonia_metallidurans_CH34_
YP_578788__Nitrobacter_hamburgensis_X14_
NP_767149__Bradyrhizobium_japonicum_
YP_530139__Rhodopseudomonas_palustris..._
NP_105559__Mesorhizobium_loti_
NP_435326__Sinorhizobium_meliloti_
YP_468459__Rhizobium_etli_CFN_42_
YP_165111__Silicibacter_pomeroyi_DSS-3_
YP_354126__Rhodobacter_sphaeroides_2_4_1_
YP_511061__Jannaschia_CCS1_
YP_422947__Magnetospirillum_magneticu..._
F41C3.3.2__Celegans_
ZP_01460913__Stigmatella_aurantiaca_
YP_635261__Myxococcus_xanthus_DK_1622_
YP_644629__Rubrobacter_xylanophilus_D..._
YP_421963__Magnetospirillum_magneticu..._
YP_366982__Burkholderia_383_
NP_882947__Bordetella_parapertussis_
YP_585941__Ralstonia_metallidurans_CH34_
0.2

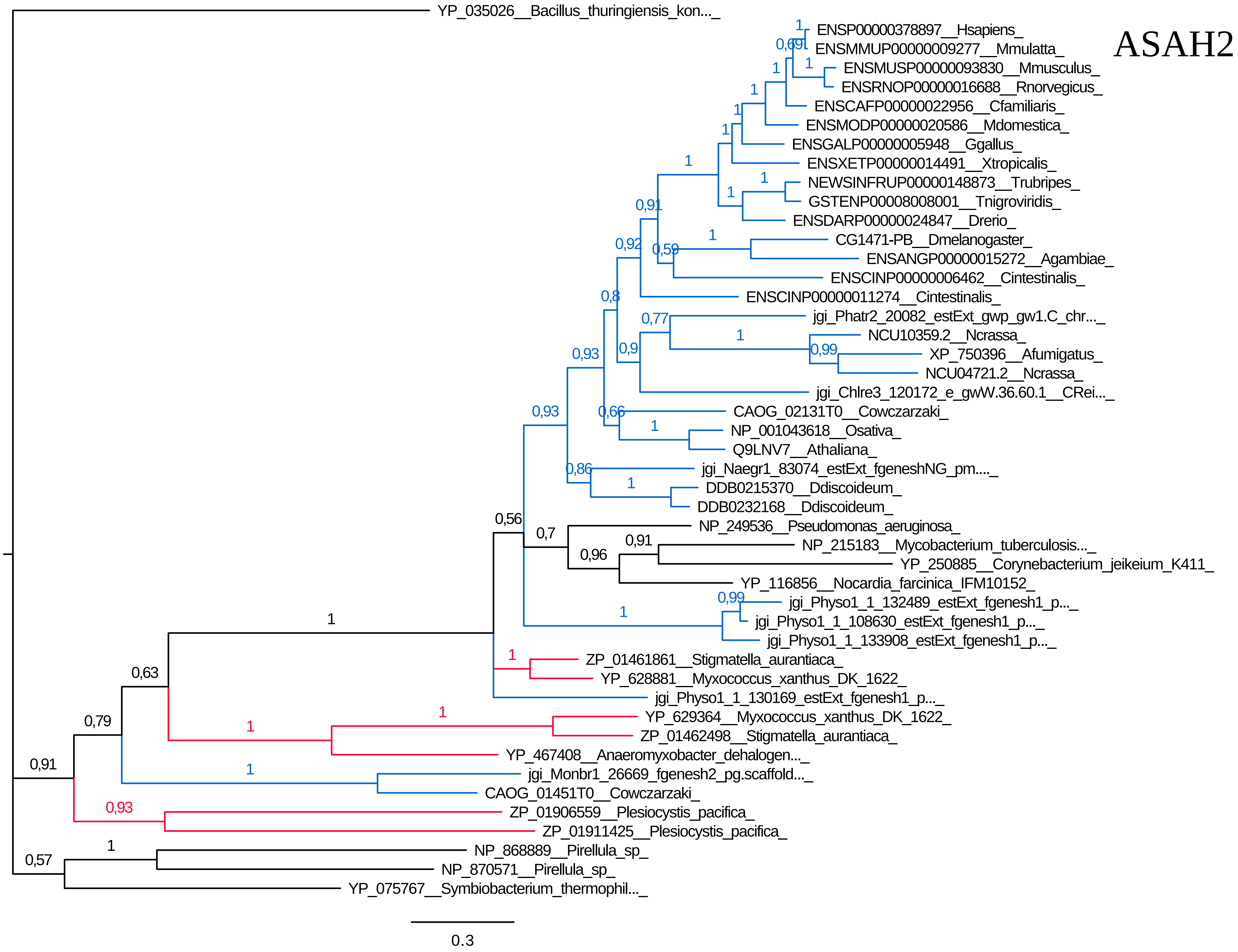
ASAH2
YP_035026__Bacillus_thuringiensis_kon..._
ENSP00000378897__Hsapiens_
ENSMMUP00000009277__Mmulatta_
ENSMUSP00000093830__Mmusculus_
ENSRNOP00000016688__Rnorvegicus_
ENSCAFP00000022956__Cfamiliaris_
ENSMODP00000020586__Mdomestica_
ENSGALP00000005948__Ggallus_
ENSXETP00000014491__Xtropicalis_
NEWSINFRUP00000148873__Trubripes_
GSTENP00008008001__Tnigroviridis_
ENSDARP00000024847__Drerio_
CG1471-PB__Dmelanogaster_
ENSANGP00000015272__Agambiae_
ENSCINP00000006462__Cintestinalis_
ENSCINP00000011274__Cintestinalis_
jgi_Phatr2_20082_estExt_gwp_gw1.C_chr..._
NCU10359.2__Ncrassa_
XP_750396__Afumigatus_
NCU04721.2__Ncrassa_
jgi_Chlre3_120172_e_gwW.36.60.1__CRei..._
CAOG_02131T0__Cowczarzaki_
NP_001043618__Osativa_
Q9LNV7__Athaliana_
jgi_Naegr1_83074_estExt_fgeneshNG_pm...._
DDB0215370__Ddiscoideum_
DDB0232168__Ddiscoideum_
NP_249536__Pseudomonas_aeruginosa_
NP_215183__Mycobacterium_tuberculosis..._
YP_250885__Corynebacterium_jeikeium_K411_
YP_116856__Nocardia_farcinica_IFM10152_
jgi_Physo1_1_132489_estExt_fgenesh1_p..._
jgi_Physo1_1_108630_estExt_fgenesh1_p..._
jgi_Physo1_1_133908_estExt_fgenesh1_p..._
ZP_01461861__Stigmatella_aurantiaca_
YP_628881__Myxococcus_xanthus_DK_1622_
jgi_Physo1_1_130169_estExt_fgenesh1_p..._
YP_629364__Myxococcus_xanthus_DK_1622_
ZP_01462498__Stigmatella_aurantiaca_
YP_467408__Anaeromyxobacter_dehalogen..._
jgi_Monbr1_26669_fgenesh2_pg.scaffold..._
CAOG_01451T0__Cowczarzaki_
ZP_01906559__Plesiocystis_pacifica_
ZP_01911425__Plesiocystis_pacifica_
NP_868889__Pirellula_sp_
NP_870571__Pirellula_sp_
YP_075767__Symbiobacterium_thermophil..._
0.3

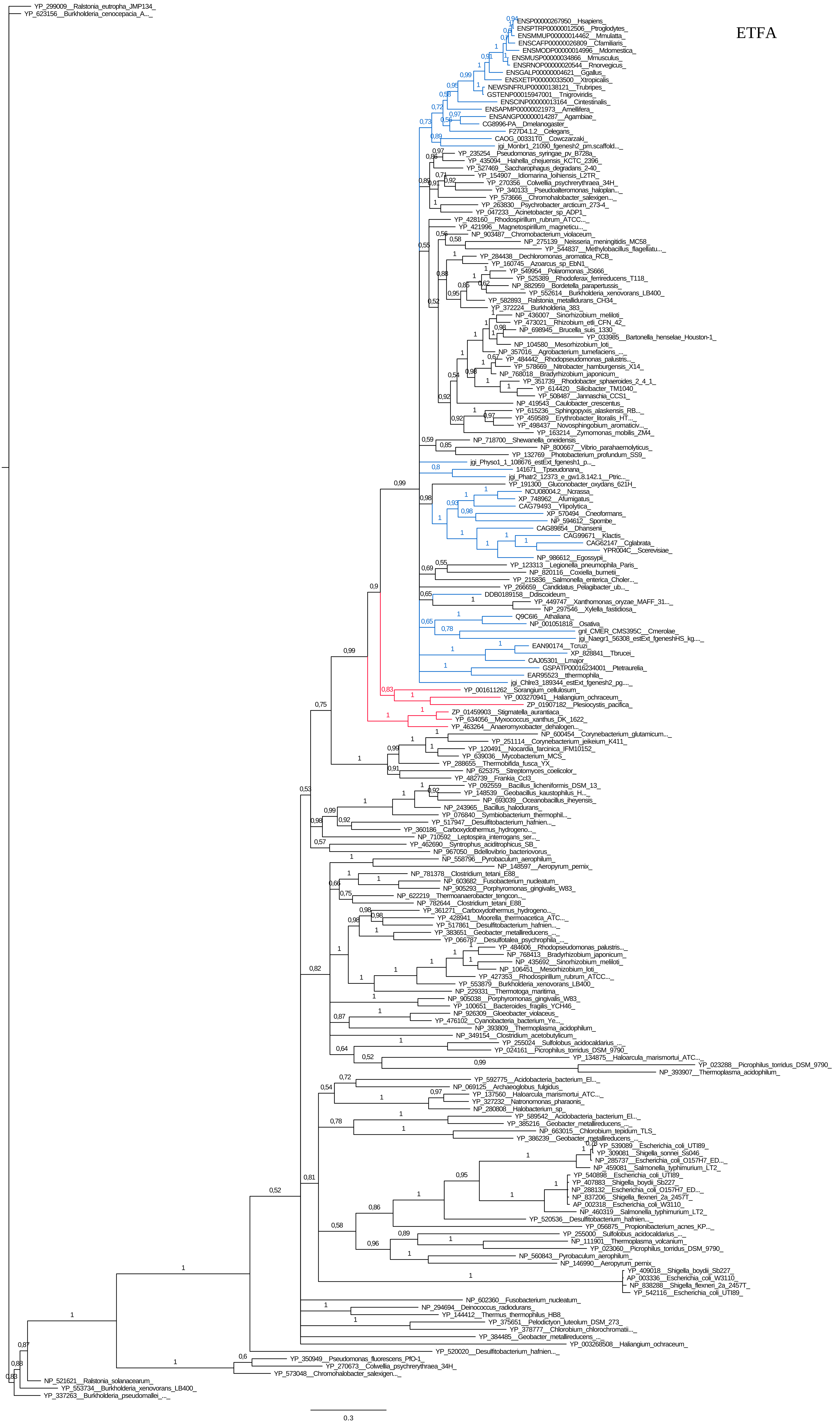
ETFA
YP_299009__Ralstonia_eutropha_JMP134_
YP_623156__Burkholderia_cenocepacia_A..._
ENSP00000267950__Hsapiens_
ENSPTRP00000012506__Ptroglodytes_
ENSMMUP00000014462__Mmulatta_
ENSCAFP00000026809__Cfamiliaris_
ENSMODP00000014996__Mdomestica_
ENSMUSP00000034866__Mmusculus_
ENSRNOP00000020544__Rnorvegicus_
ENSGALP00000004621__Ggallus_
ENSXETP00000033500__Xtropicalis_
NEWSINFRUP00000138121__Trubripes_
GSTENP00015947001__Tnigroviridis_
ENSCINP00000013164__Cintestinalis_
ENSAPMP00000021973__Amellifera_
ENSANGP00000014287__Agambiae_
CG8996-PA__Dmelanogaster_
F27D4.1.2__Celegans_
CAOG_00331T0__Cowczarzaki_
jgi_Monbr1_21090_fgenesh2_pm.scaffold..._
YP_235254__Pseudomonas_syringae_pv_B728a_
YP_435094__Hahella_chejuensis_KCTC_2396_
YP_527469__Saccharophagus_degradans_2-40_
YP_154907__Idiomarina_loihiensis_L2TR_
YP_270356__Colwellia_psychrerythraea_34H_
YP_340133__Pseudoalteromonas_haloplan..._
YP_573666__Chromohalobacter_salexigen..._
YP_263830__Psychrobacter_arcticum_273-4_
YP_047233__Acinetobacter_sp_ADP1_
YP_428160__Rhodospirillum_rubrum_ATCC..._
YP_421996__Magnetospirillum_magneticu..._
NP_903487__Chromobacterium_violaceum_
NP_275139__Neisseria_meningitidis_MC58_
YP_544837__Methylobacillus_flagellatu..._
YP_284438__Dechloromonas_aromatica_RCB_
YP_160745__Azoarcus_sp_EbN1_
YP_549954__Polaromonas_JS666_
YP_525389__Rhodoferax_ferrireducens_T118_
NP_882959__Bordetella_parapertussis_
YP_552614__Burkholderia_xenovorans_LB400_
YP_582893__Ralstonia_metallidurans_CH34_
YP_372224__Burkholderia_383_
NP_436007__Sinorhizobium_meliloti_
YP_473021__Rhizobium_etli_CFN_42_
NP_698945__Brucella_suis_1330_
YP_033985__Bartonella_henselae_Houston-1_
NP_104580__Mesorhizobium_loti_
NP_357016__Agrobacterium_tumefaciens_..._
YP_484442__Rhodopseudomonas_palustris..._
YP_578669__Nitrobacter_hamburgensis_X14_
NP_768018__Bradyrhizobium_japonicum_
YP_351739__Rhodobacter_sphaeroides_2_4_1_
YP_614420__Silicibacter_TM1040_
YP_508487__Jannaschia_CCS1_
NP_419543__Caulobacter_crescentus_
YP_615236__Sphingopyxis_alaskensis_RB..._
YP_459589__Erythrobacter_litoralis_HT..._
YP_498437__Novosphingobium_aromaticiv..._
YP_163214__Zymomonas_mobilis_ZM4_
NP_718700__Shewanella_oneidensis_
NP_800667__Vibrio_parahaemolyticus_
YP_132769__Photobacterium_profundum_SS9_
jgi_Physo1_1_108676_estExt_fgenesh1_p..._
141671__Tpseudonana_
jgi_Phatr2_12373_e_gw1.8.142.1__Ptric..._
YP_191300__Gluconobacter_oxydans_621H_
NCU08004.2__Ncrassa_
XP_748962__Afumigatus_
CAG79493__Ylipolytica_
XP_570494__Cneoformans_
NP_594612__Spombe_
CAG89854__Dhansenii_
CAG99671__Klactis_
CAG62147__Cglabrata_
YPR004C__Scerevisiae_
NP_986612__Egossypii_
YP_123313__Legionella_pneumophila_Paris_
NP_820116__Coxiella_burnetii_
YP_215836__Salmonella_enterica_Choler..._
YP_266659__Candidatus_Pelagibacter_ub..._
DDB0189158__Ddiscoideum_
YP_449747__Xanthomonas_oryzae_MAFF_31..._
NP_297546__Xylella_fastidiosa_
Q9C6I6__Athaliana_
NP_001051818__Osativa_
gnl_CMER_CMS395C__Cmerolae_
jgi_Naegr1_56308_estExt_fgeneshHS_kg..._
EAN90174__Tcruzi_
XP_828841__Tbrucei_
CAJ05301__Lmajor_
GSPATP00016234001__Ptetraurelia_
EAR95523__tthermophila_
jgi_Chlre3_189344_estExt_fgenesh2_pg..._
YP_001611262__Sorangium_cellulosum_
YP_003270941__Haliangium_ochraceum_
ZP_01907182__Plesiocystis_pacifica_
ZP_01459903__Stigmatella_aurantiaca_
YP_634056__Myxococcus_xanthus_DK_1622_
YP_463264__Anaeromyxobacter_dehalogen..._
NP_600454__Corynebacterium_glutamicum..._
YP_251114__Corynebacterium_jeikeium_K411_
YP_120491__Nocardia_farcinica_IFM10152_
YP_639036__Mycobacterium_MCS_
YP_288655__Thermobifida_fusca_YX_
NP_625375__Streptomyces_coelicolor_
YP_482739__Frankia_CcI3_
YP_092559__Bacillus_licheniformis_DSM_13_
YP_148539__Geobacillus_kaustophilus_H..._
NP_693039__Oceanobacillus_iheyensis_
NP_243965__Bacillus_halodurans_
YP_076780__Symbiobacterium_thermophil..._
YP_517947__Desulfitobacterium_hafnien..._
YP_360186__Carboxydothermus_hydrogeno..._
NP_710592__Leptospira_interrogans_ser..._
YP_462690__Syntrophus_aciditrophicus_SB_
NP_967050__Bdellovibrio_bacteriovorus_
NP_558796__Pyrobaculum_aerophilum_
NP_148597__Aeropyrum_pernix_
NP_781378__Clostridium_tetani_E88_
NP_603682__Fusobacterium_nucleatum_
NP_905293__Porphyromonas_gingivalis_W83_
NP_622219__Thermoanaerobacter_tengcon..._
NP_782644__Clostridium_tetani_E88_
YP_361271__Carboxydothermus_hydrogeno..._
YP_428941__Moorella_thermoacetica_ATC..._
YP_517861__Desulfitobacterium_hafnien..._
YP_383651__Geobacter_metallireducens_..._
YP_066787__Desulfotalea_psychrophila_..._
YP_484606__Rhodopseudomonas_palustris..._
NP_768413__Bradyrhizobium_japonicum_
NP_435692__Sinorhizobium_meliloti_
NP_106451__Mesorhizobium_loti_
YP_427353__Rhodospirillum_rubrum_ATCC..._
YP_553879__Burkholderia_xenovorans_LB400_
NP_229331__Thermotoga_maritima_
NP_905038__Porphyromonas_gingivalis_W83_
YP_100651__Bacteroides_fragilis_YCH46_
NP_926309__Gloeobacter_violaceus_
YP_476102__Cyanobacteria_bacterium_Ye..._
NP_393809__Thermoplasma_acidophilum_
NP_349154__Clostridium_acetobutylicum_
YP_255024__Sulfolobus_acidocaldarius_..._
YP_024161__Picrophilus_torridus_DSM_9790_
YP_134875__Haloarcula_marismortui_ATC..._
YP_023288__Picrophilus_torridus_DSM_9790_
NP_393907__Thermoplasma_acidophilum_
YP_592775__Acidobacteria_bacterium_El..._
NP_069125__Archaeoglobus_fulgidus_
YP_137560__Haloarcula_marismortui_ATC..._
YP_327232__Natronomonas_pharaonis_
NP_280808__Halobacterium_sp_
YP_589542__Acidobacteria_bacterium_El..._
YP_385216__Geobacter_metallireducens_..._
NP_663015__Chlorobium_tepidum_TLS_
YP_386239__Geobacter_metallireducens_..._
YP_539089__Escherichia_coli_UTI89_
YP_309081__Shigella_sonnei_Ss046_
NP_285737__Escherichia_coli_O157H7_ED..._
NP_459081__Salmonella_typhimurium_LT2_
YP_540898__Escherichia_coli_UTI89_
YP_407883__Shigella_boydii_Sb227_
NP_288132__Escherichia_coli_O157H7_ED..._
NP_837206__Shigella_flexneri_2a_2457T_
AP_002318__Escherichia_coli_W3110_
NP_460319__Salmonella_typhimurium_LT2_
YP_520536__Desulfitobacterium_hafnien..._
YP_056875__Propionibacterium_acnes_KP..._
YP_255000__Sulfolobus_acidocaldarius_..._
NP_111901__Thermoplasma_volcanium_
YP_023060__Picrophilus_torridus_DSM_9790_
NP_560843__Pyrobaculum_aerophilum_
NP_146990__Aeropyrum_pernix_
YP_409018__Shigella_boydii_Sb227_
AP_003336__Escherichia_coli_W3110_
NP_838288__Shigella_flexneri_2a_2457T_
YP_542116__Escherichia_coli_UTI89_
NP_602360__Fusobacterium_nucleatum_
NP_294694__Deinococcus_radiodurans_
YP_144412__Thermus_thermophilus_HB8_
YP_375651__Pelodictyon_luteolum_DSM_273_
YP_378777__Chlorobium_chlorochromatii..._
YP_384485__Geobacter_metallireducens_..._
YP_003268508__Haliangium_ochraceum_
YP_520020__Desulfitobacterium_hafnien..._
YP_350949__Pseudomonas_fluorescens_PfO-1_
YP_270673__Colwellia_psychrerythraea_34H_
YP_573048__Chromohalobacter_salexigen..._
NP_521621__Ralstonia_solanacearum_
YP_553734__Burkholderia_xenovorans_LB400_
YP_337263__Burkholderia_pseudomallei_..._
0.3

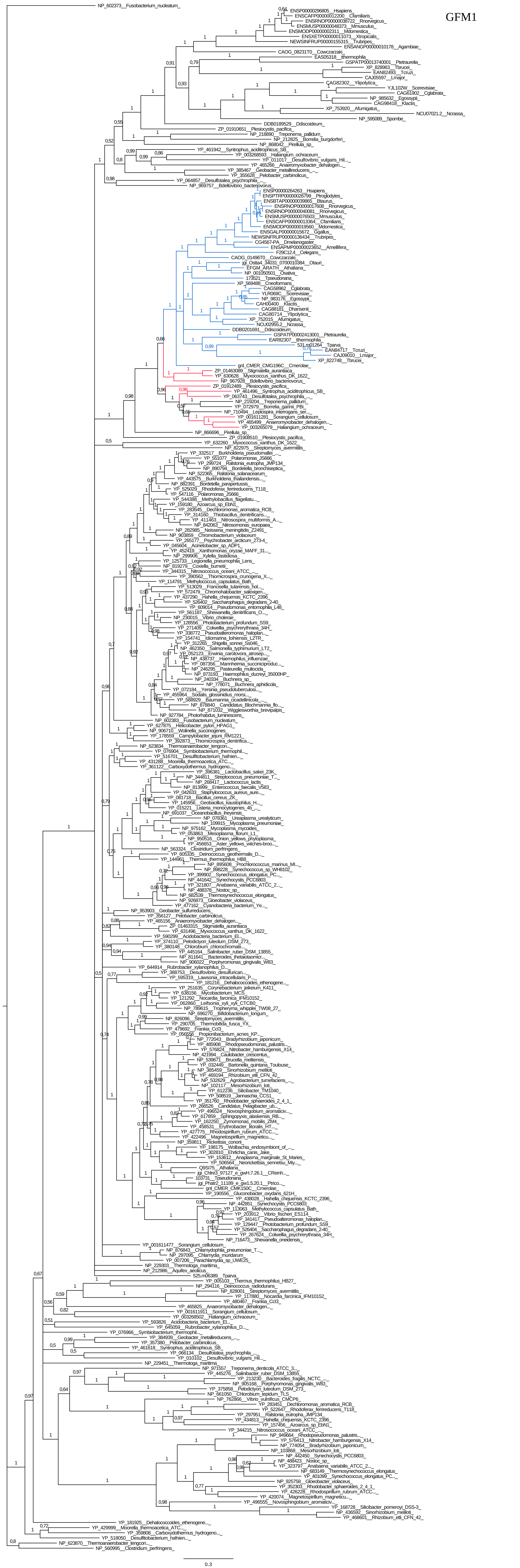
GFM1
0.3

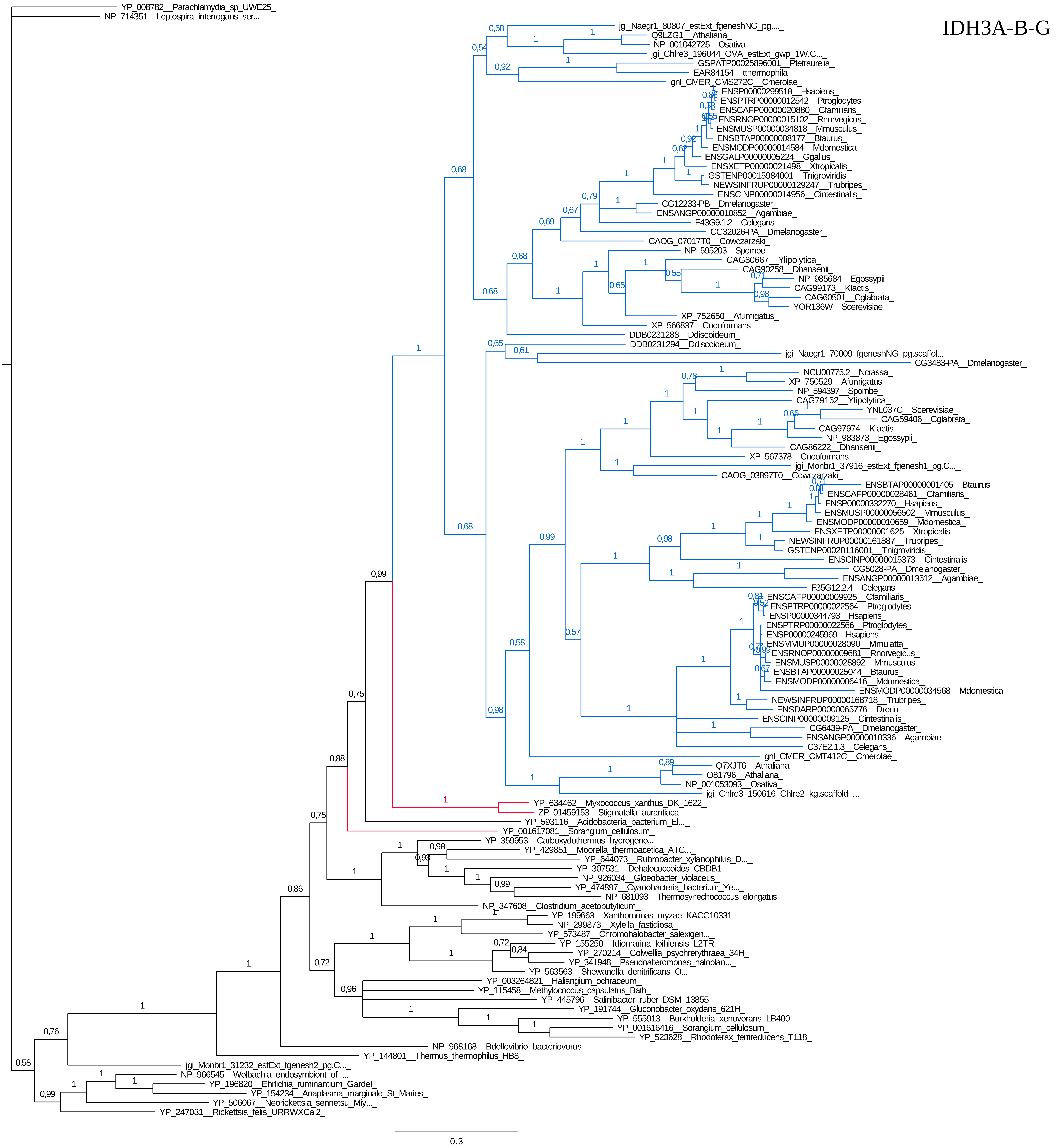
IDH3A-B-G
YP_008782__Parachlamydia_sp_UWE25_
NP_714351__Leptospira_interrogans_ser..._
jgi_Naegr1_80807_estExt_fgeneshNG_pg...._
Q9LZG1__Athaliana_
NP_001042725__Osativa_
jgi_Chlre3_196044_OVA_estExt_gwp_1W.C..._
GSPATP00025896001__Ptetraurelia_
EAR84154__tthermophila_
gnl_CMER_CMS272C__Cmerolae_
ENSP00000299518__Hsapiens_
ENSPTRP00000012542__Ptroglodytes_
ENSCAFP00000020880__Cfamiliaris_
ENSRNOP00000015102__Rnorvegicus_
ENSMUSP00000034818__Mmusculus_
ENSBTAP00000008177__Btaurus_
ENSMODP00000014584__Mdomestica_
ENSGALP00000005224__Ggallus_
ENSXETP00000021498__Xtropicalis_
GSTENP00015984001__Tnigroviridis_
NEWSINFRUP00000129247__Trubripes_
ENSCINP00000014956__Cintestinalis_
CG12233-PB__Dmelanogaster_
ENSANGP00000010852__Agambiae_
F43G9.1.2__Celegans_
CG32026-PA__Dmelanogaster_
CAOG_07017T0__Cowczarzaki_
NP_595203__Spombe_
CAG80667__Ylipolytica_
CAG90258__Dhansenii_
NP_985684__Egossypii_
CAG99173__Klactis_
CAG60501__Cglabrata_
YOR136W__Scerevisiae_
XP_752650__Afumigatus_
XP_566837__Cneoformans_
DDB0231288__Ddiscoideum_
DDB0231294__Ddiscoideum_
jgi_Naegr1_70009_fgeneshNG_pg.scaffol..._
CG3483-PA__Dmelanogaster_
NCU00775.2__Ncrassa_
XP_750529__Afumigatus_
NP_594397__Spombe_
CAG79152__Ylipolytica_
YNL037C__Scerevisiae_
CAG59406__Cglabrata_
CAG97974__Klactis_
NP_983873__Egossypii_
CAG86222__Dhansenii_
XP_567378__Cneoformans_
jgi_Monbr1_37916_estExt_fgenesh1_pg.C..._
CAOG_03897T0__Cowczarzaki_
ENSBTAP00000001405__Btaurus_
ENSCAFP00000028461__Cfamiliaris_
ENSP00000332270__Hsapiens_
ENSMUSP00000056502__Mmusculus_
ENSMODP00000010659__Mdomestica_
ENSXETP00000001625__Xtropicalis_
NEWSINFRUP00000161887__Trubripes_
GSTENP00028116001__Tnigroviridis_
ENSCINP00000015373__Cintestinalis_
CG5028-PA__Dmelanogaster_
ENSANGP00000013512__Agambiae_
F35G12.2.4__Celegans_
ENSCAFP00000009925__Cfamiliaris_
ENSPTRP00000022564__Ptroglodytes_
ENSP00000344793__Hsapiens_
ENSPTRP00000022566__Ptroglodytes_
ENSP00000245969__Hsapiens_
ENSMMUP00000028090__Mmulatta_
ENSRNOP00000009681__Rnorvegicus_
ENSMUSP00000028892__Mmusculus_
ENSBTAP00000025044__Btaurus_
ENSMODP00000006416__Mdomestica_
ENSMODP00000034568__Mdomestica_
NEWSINFRUP00000168718__Trubripes_
ENSDARP00000065776__Drerio_
ENSCINP00000009125__Cintestinalis_
CG6439-PA__Dmelanogaster_
ENSANGP00000010336__Agambiae_
C37E2.1.3__Celegans_
gnl_CMER_CMT412C__Cmerolae_
Q7XJT6__Athaliana_
O81796__Athaliana_
NP_001053093__Osativa_
jgi_Chlre3_150616_Chlre2_kg.scaffold_..._
YP_634462__Myxococcus_xanthus_DK_1622_
ZP_01459153__Stigmatella_aurantiaca_
YP_593116__Acidobacteria_bacterium_El..._
YP_001617081__Sorangium_cellulosum_
YP_359953__Carboxydothermus_hydrogeno..._
YP_429851__Moorella_thermoacetica_ATC..._
YP_644073__Rubrobacter_xylanophilus_D..._
YP_307531__Dehalococcoides_CBDB1_
NP_926034__Gloeobacter_violaceus_
YP_474897__Cyanobacteria_bacterium_Ye..._
NP_681093__Thermosynechococcus_elongatus_
NP_347608__Clostridium_acetobutylicum_
YP_199663__Xanthomonas_oryzae_KACC10331_
NP_299873__Xylella_fastidiosa_
YP_573487__Chromohalobacter_salexigen..._
YP_155250__Idiomarina_loihiensis_L2TR_
YP_270214__Colwellia_psychrerythraea_34H_
YP_341948__Pseudoalteromonas_haloplan..._
YP_563563__Shewanella_denitrificans_O..._
YP_003264821__Haliangium_ochraceum_
YP_115458__Methylococcus_capsulatus_Bath_
YP_445796__Salinibacter_ruber_DSM_13855_
YP_191744__Gluconobacter_oxydans_621H_
YP_555913__Burkholderia_xenovorans_LB400_
YP_001616416__Sorangium_cellulosum_
YP_523628__Rhodoferax_ferrireducens_T118_
NP_968168__Bdellovibrio_bacteriovorus_
YP_144801__Thermus_thermophilus_HB8_
jgi_Monbr1_31232_estExt_fgenesh2_pg.C..._
NP_966545__Wolbachia_endosymbiont_of_..._
YP_196820__Ehrlichia_ruminantium_Gardel_
YP_154234__Anaplasma_marginale_St_Maries_
YP_506067__Neorickettsia_sennetsu_Miy..._
YP_247031__Rickettsia_felis_URRWXCal2_
0.3

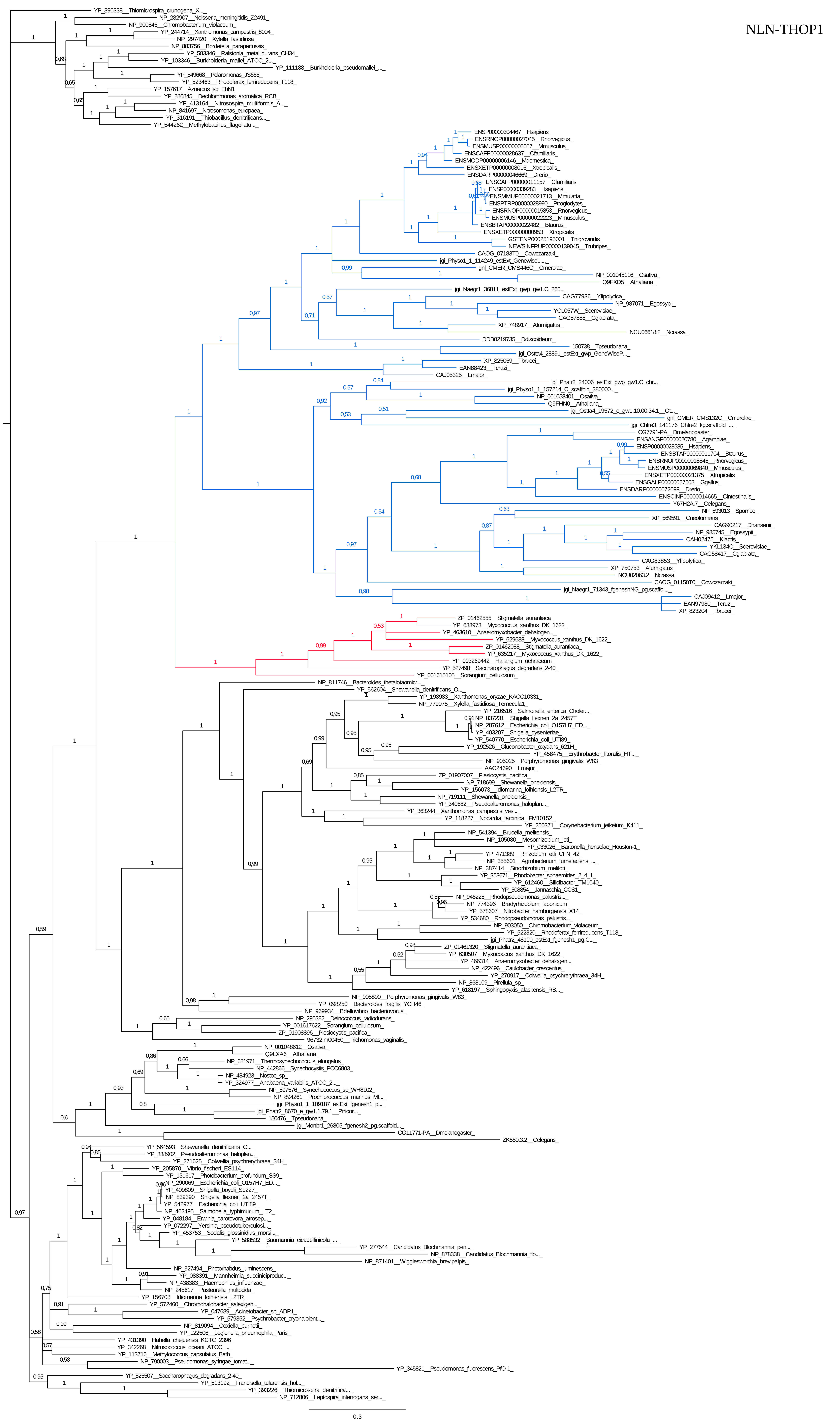
NLN-THOP1
YP_390338__Thiomicrospira_crunogena_X..._
NP_282907__Neisseria_meningitidis_Z2491_
NP_900546__Chromobacterium_violaceum_
YP_244714__Xanthomonas_campestris_8004_
NP_297420__Xylella_fastidiosa_
NP_883756__Bordetella_parapertussis_
YP_583346__Ralstonia_metallidurans_CH34_
YP_103346__Burkholderia_mallei_ATCC_2..._
YP_111188__Burkholderia_pseudomallei_..._
YP_549668__Polaromonas_JS666_
YP_523463__Rhodoferax_ferrireducens_T118_
YP_157617__Azoarcus_sp_EbN1_
YP_286845__Dechloromonas_aromatica_RCB_
YP_413164__Nitrosospira_multiformis_A..._
NP_841697__Nitrosomonas_europaea_
YP_316191__Thiobacillus_denitrificans..._
YP_544262__Methylobacillus_flagellatu..._
ENSP00000304467__Hsapiens_
ENSRNOP00000027045__Rnorvegicus_
ENSMUSP00000005057__Mmusculus_
ENSCAFP00000028637__Cfamiliaris_
ENSMODP00000006146__Mdomestica_
ENSXETP00000008016__Xtropicalis_
ENSDARP00000046669__Drerio_
ENSCAFP00000011157__Cfamiliaris_
ENSP00000339283__Hsapiens_
ENSMMUP00000021713__Mmulatta_
ENSPTRP00000028990__Ptroglodytes_
ENSRNOP00000015853__Rnorvegicus_
ENSMUSP00000022223__Mmusculus_
ENSBTAP00000022482__Btaurus_
ENSXETP00000000953__Xtropicalis_
GSTENP00025195001__Tnigroviridis_
NEWSINFRUP00000139045__Trubripes_
CAOG_07183T0__Cowczarzaki_
jgi_Physo1_1_114249_estExt_Genewise1..._
gnl_CMER_CMS446C__Cmerolae_
NP_001045116__Osativa_
Q9FXD5__Athaliana_
jgi_Naegr1_36811_estExt_gwp_gw1.C_260..._
CAG77936__Ylipolytica_
NP_987071__Egossypii_
YCL057W__Scerevisiae_
CAG57888__Cglabrata_
XP_748917__Afumigatus_
NCU06618.2__Ncrassa_
DDB0219735__Ddiscoideum_
150738__Tpseudonana_
jgi_Ostta4_28891_estExt_gwp_GeneWiseP..._
XP_825059__Tbrucei_
EAN88423__Tcruzi_
CAJ05325__Lmajor_
jgi_Phatr2_24006_estExt_gwp_gw1.C_chr..._
jgi_Physo1_1_157214_C_scaffold_380000..._
NP_001058401__Osativa_
Q9FHN0__Athaliana_
jgi_Ostta4_19572_e_gw1.10.00.34.1__Ot..._
gnl_CMER_CMS132C__Cmerolae_
jgi_Chlre3_141176_Chlre2_kg.scaffold_..._
CG7791-PA__Dmelanogaster_
ENSANGP00000020780__Agambiae_
ENSP00000028585__Hsapiens_
ENSBTAP00000011704__Btaurus_
ENSRNOP00000018845__Rnorvegicus_
ENSMUSP00000069840__Mmusculus_
ENSXETP00000021375__Xtropicalis_
ENSGALP00000027603__Ggallus_
ENSDARP00000072099__Drerio_
ENSCINP00000014665__Cintestinalis_
Y67H2A.7__Celegans_
NP_593013__Spombe_
XP_569591__Cneoformans_
CAG90217__Dhansenii_
NP_985745__Egossypii_
CAH02475__Klactis_
YKL134C__Scerevisiae_
CAG58417__Cglabrata_
CAG83853__Ylipolytica_
XP_750753__Afumigatus_
NCU02063.2__Ncrassa_
CAOG_01150T0__Cowczarzaki_
jgi_Naegr1_71343_fgeneshNG_pg.scaffol..._
CAJ09412__Lmajor_
EAN97980__Tcruzi_
XP_823204__Tbrucei_
ZP_01462555__Stigmatella_aurantiaca_
YP_633973__Myxococcus_xanthus_DK_1622_
YP_463610__Anaeromyxobacter_dehalogen..._
YP_629638__Myxococcus_xanthus_DK_1622_
ZP_01462088__Stigmatella_aurantiaca_
YP_635217__Myxococcus_xanthus_DK_1622_
YP_003269442__Haliangium_ochraceum_
YP_527498__Saccharophagus_degradans_2-40_
YP_001615105__Sorangium_cellulosum_
NP_811746__Bacteroides_thetaiotaomicr..._
YP_562604__Shewanella_denitrificans_O..._
YP_198983__Xanthomonas_oryzae_KACC10331_
NP_779075__Xylella_fastidiosa_Temecula1_
YP_216516__Salmonella_enterica_Choler..._
NP_837231__Shigella_flexneri_2a_2457T_
NP_287612__Escherichia_coli_O157H7_ED..._
YP_403207__Shigella_dysenteriae_
YP_540770__Escherichia_coli_UTI89_
YP_192526__Gluconobacter_oxydans_621H_
YP_458475__Erythrobacter_litoralis_HT..._
NP_905025__Porphyromonas_gingivalis_W83_
AAC24690__Lmajor_
ZP_01907007__Plesiocystis_pacifica_
NP_718699__Shewanella_oneidensis_
YP_156073__Idiomarina_loihiensis_L2TR_
NP_719111__Shewanella_oneidensis_
YP_340682__Pseudoalteromonas_haloplan..._
YP_363244__Xanthomonas_campestris_ves..._
YP_118227__Nocardia_farcinica_IFM10152_
YP_250371__Corynebacterium_jeikeium_K411_
NP_541394__Brucella_melitensis_
NP_105080__Mesorhizobium_loti_
YP_033026__Bartonella_henselae_Houston-1_
YP_471389__Rhizobium_etli_CFN_42_
NP_355601__Agrobacterium_tumefaciens_..._
NP_387414__Sinorhizobium_meliloti_
YP_353671__Rhodobacter_sphaeroides_2_4_1_
YP_612460__Silicibacter_TM1040_
YP_508854__Jannaschia_CCS1_
NP_946225__Rhodopseudomonas_palustris..._
NP_774396__Bradyrhizobium_japonicum_
YP_578607__Nitrobacter_hamburgensis_X14_
YP_534680__Rhodopseudomonas_palustris..._
NP_903050__Chromobacterium_violaceum_
YP_522320__Rhodoferax_ferrireducens_T118_
jgi_Phatr2_48190_estExt_fgenesh1_pg.C..._
ZP_01461320__Stigmatella_aurantiaca_
YP_630507__Myxococcus_xanthus_DK_1622_
YP_466314__Anaeromyxobacter_dehalogen..._
NP_422496__Caulobacter_crescentus_
YP_270917__Colwellia_psychrerythraea_34H_
NP_868109__Pirellula_sp_
YP_618197__Sphingopyxis_alaskensis_RB..._
NP_905890__Porphyromonas_gingivalis_W83_
YP_098250__Bacteroides_fragilis_YCH46_
NP_969934__Bdellovibrio_bacteriovorus_
NP_295382__Deinococcus_radiodurans_
YP_001617622__Sorangium_cellulosum_
ZP_01908896__Plesiocystis_pacifica_
96732.m00450__Trichomonas_vaginalis_
NP_001048612__Osativa_
Q9LXA6__Athaliana_
NP_681971__Thermosynechococcus_elongatus_
NP_442866__Synechocystis_PCC6803_
NP_484923__Nostoc_sp_
YP_324977__Anabaena_variabilis_ATCC_2..._
NP_897576__Synechococcus_sp_WH8102_
NP_894261__Prochlorococcus_marinus_MI..._
jgi_Physo1_1_109187_estExt_fgenesh1_p..._
jgi_Phatr2_8670_e_gw1.1.79.1__Ptricor..._
150476__Tpseudonana_
jgi_Monbr1_26805_fgenesh2_pg.scaffold..._
CG11771-PA__Dmelanogaster_
ZK550.3.2__Celegans_
YP_564593__Shewanella_denitrificans_O..._
YP_338902__Pseudoalteromonas_haloplan..._
YP_271625__Colwellia_psychrerythraea_34H_
YP_205870__Vibrio_fischeri_ES114_
YP_131617__Photobacterium_profundum_SS9_
NP_290069__Escherichia_coli_O157H7_ED..._
YP_409809__Shigella_boydii_Sb227_
NP_839390__Shigella_flexneri_2a_2457T_
YP_542977__Escherichia_coli_UTI89_
NP_462495__Salmonella_typhimurium_LT2_
YP_048184__Erwinia_carotovora_atrosep..._
YP_072297__Yersinia_pseudotuberculosi..._
YP_453753__Sodalis_glossinidius_morsi..._
YP_588532__Baumannia_cicadellinicola_..._
YP_277544__Candidatus_Blochmannia_pen..._
NP_878338__Candidatus_Blochmannia_flo..._
NP_871401__Wigglesworthia_brevipalpis_
NP_927494__Photorhabdus_luminescens_
YP_088391__Mannheimia_succiniciproduc..._
NP_438383__Haemophilus_influenzae_
NP_245617__Pasteurella_multocida_
YP_156708__Idiomarina_loihiensis_L2TR_
YP_572460__Chromohalobacter_salexigen..._
YP_047689__Acinetobacter_sp_ADP1_
YP_579352__Psychrobacter_cryohalolent..._
NP_819094__Coxiella_burnetii_
YP_122506__Legionella_pneumophila_Paris_
YP_431390__Hahella_chejuensis_KCTC_2396_
YP_342268__Nitrosococcus_oceani_ATCC_..._
YP_113716__Methylococcus_capsulatus_Bath_
NP_790003__Pseudomonas_syringae_tomat..._
YP_345821__Pseudomonas_fluorescens_PfO-1_
YP_525507__Saccharophagus_degradans_2-40_
YP_513192__Francisella_tularensis_hol..._
YP_393226__Thiomicrospira_denitrifica..._
NP_712806__Leptospira_interrogans_ser..._
0.3

NPEPL1

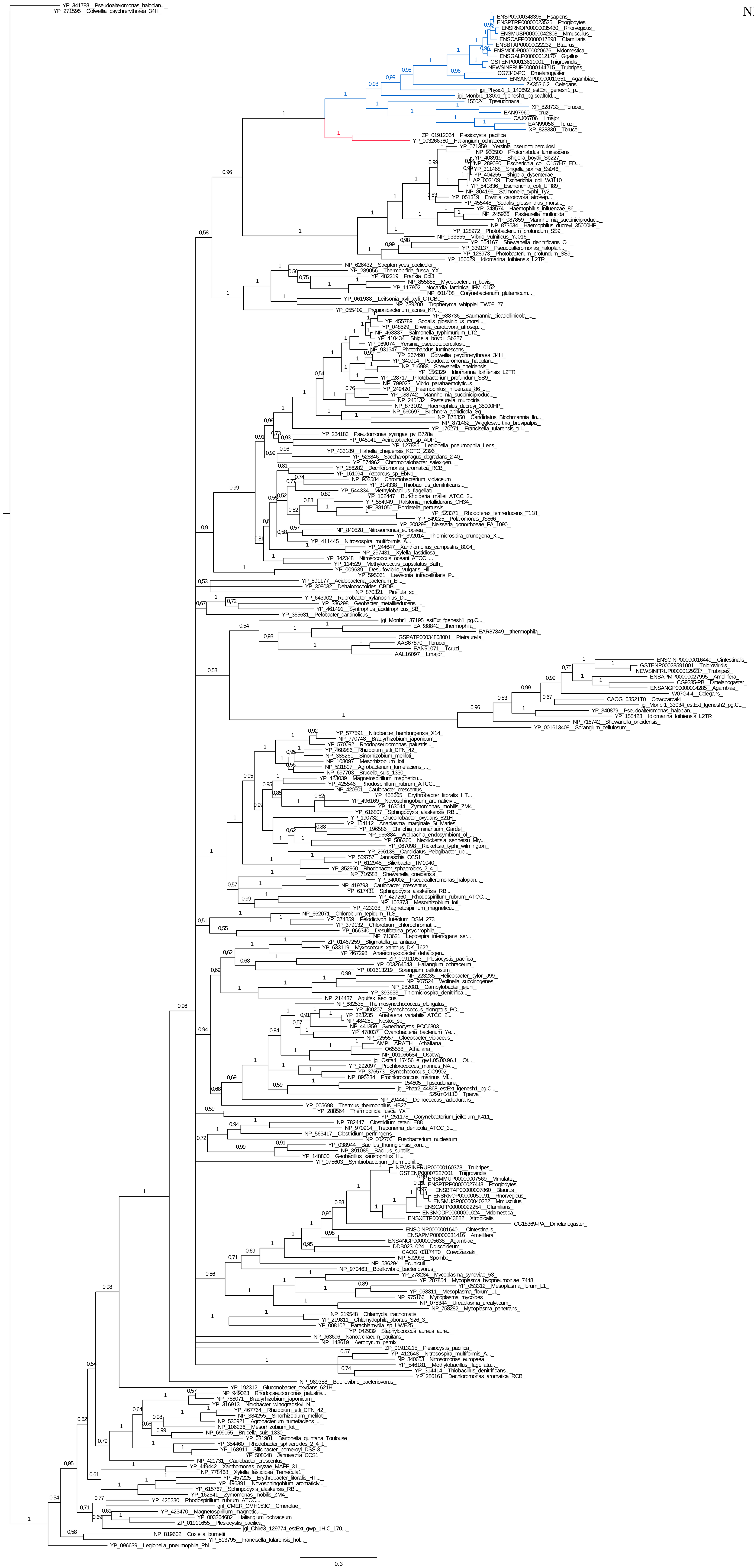

RARS2

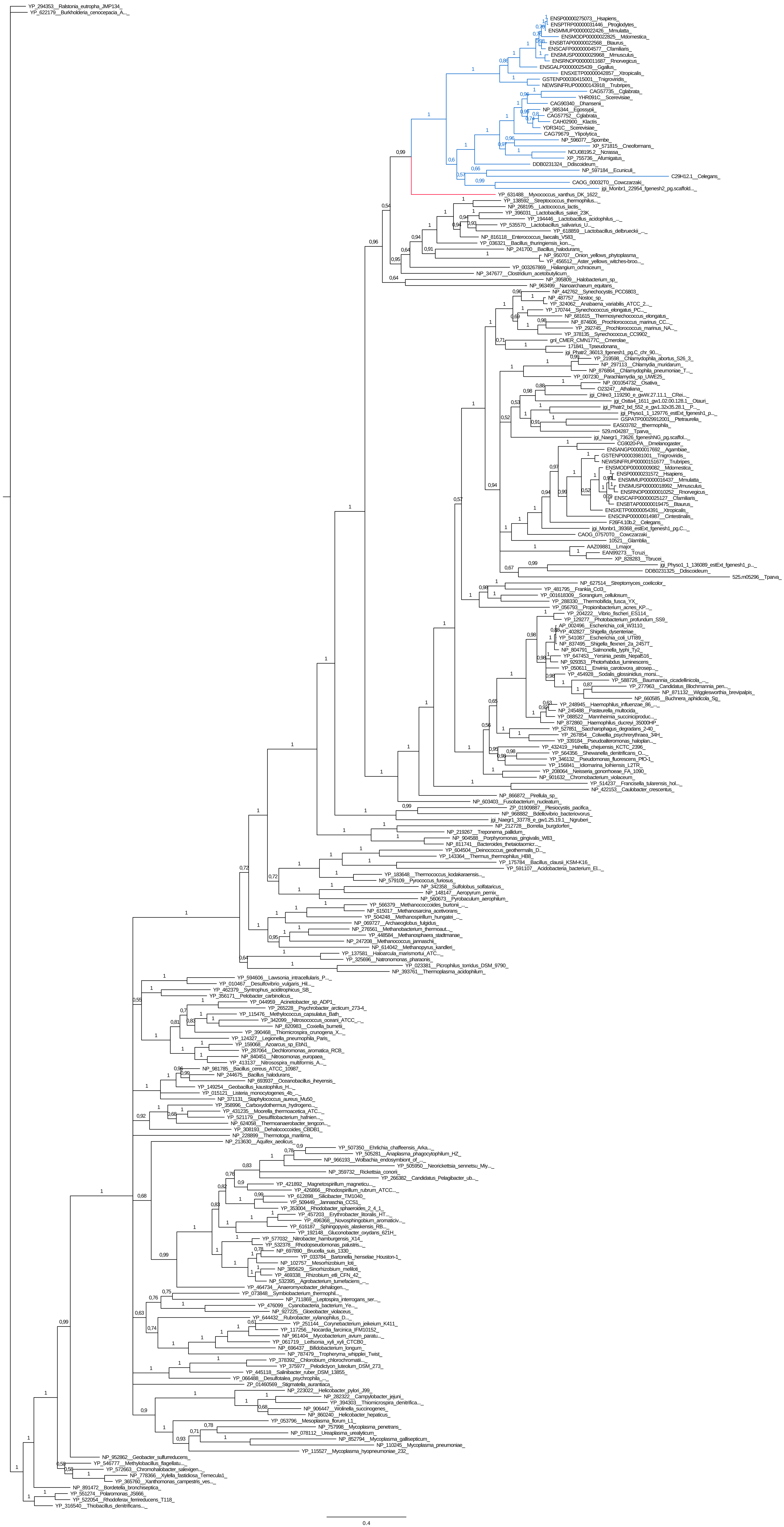

Supplement: Dataset S2 — Bayesian phylogenetic trees of the 15 eukaryotic proteins that branch with a myxococcal clade. The eukaryotic and myxococcal clades are highlighted in blue and red, respectively. (PDF) [file pone.0021989.s005.pdf]
